# Antibiofilm and Antimicrobial Activities of Chloroindoles Against Uropathogenic *Escherichia coli*

**DOI:** 10.3389/fmicb.2022.872943

**Published:** 2022-06-16

**Authors:** Bharath Reddy Boya, Jin-Hyung Lee, Jintae Lee

**Affiliations:** School of Chemical Engineering, Yeungnam University, Gyeongsan, South Korea

**Keywords:** biofilm, chloroindoles, *E. coli*, indoles, UPEC, virulence

## Abstract

Uropathogenic *Escherichia coli* (UPEC) is a nosocomial pathogen associated with urinary tract infections and expresses several virulence factors that cause recurring infections and cystitis of the bladder, which can lead to pyelonephritis. UPEC uses different types of extracellular appendages like fimbriae and pili that aid colonization and adherence to bladder epithelium and can form persistent biofilm-like bacterial communities that aid its survival after the deployment of host immune responses. We investigated the antibiofilm, antimicrobial, and antivirulence properties of three indole derivatives namely, 4-chloroindole, 5-chloroindole, and 5-chloro 2-methyl indole. All the three chloroindoles had MICs of 75 μg/ml and inhibited biofilm formation by an average of 67% at 20 μg/ml. In addition, they inhibited swarming and swimming motilities, which are essential for dissemination from bacterial communities and colonization, reduced cell surface hydrophobicity, and inhibited indole production and curli formation. Gene expression analysis showed all three chloroindoles significantly downregulated the expressions of virulence genes associated with adhesion, stress regulation, and toxin production. A 3D-QSAR analysis revealed substitutions at the fourth and fifth positions of the indole moiety favored antimicrobial activity. Furthermore, these chloroindoles potently inhibited biofilm formation in other nosocomial pathogens and polymicrobial consortia.

## Introduction

*Escherichia coli* is a diverse Gram-negative bacterial species that can sustain itself in many niche environments, especially in the mammalian gastrointestinal tract. However, some *E. coli* strains are pathogenic and colonize the urinary tract, and of these, extraintestinal pathogenic *E. coli* is referred to as uropathogenic *E. coli* (UPEC; Kaper et al., 2004). UPEC accounts for an alarming 70%–95% of urinary tract infections (UTIs) and 50% of nosocomial UTIs, and annually, the total estimated cost of community-acquired UTIs in the United States amounts to ~$1.6 billion, and UPEC contributed substantially to this cost burden (Foxman, 2003). UPEC expresses a myriad of virulence factors, including adhesins, toxins (e.g., hemolysin), iron-acquisition systems, outer membrane proteins, and surface structural components like flagella, pili, and curli (Bien et al., 2012). When UPEC colonizes the bladder, it causes cystitis of the bladder and, in more severe cases, ascends to the kidneys causing pyelonephritis (Bower et al., 2005). Immune responses triggered in human hosts include antimicrobial compounds, cytokine production, oxygen species generation, and extreme defense measures like exfoliation of bladder cells, and in turn, UPEC deploys several evasion strategies to persist in the urinary tract, such as binding to host bladder epithelium against urine flow, multiplying rapidly to form intracellular biofilm-like bacterial communities and moving deeper into underlying immature bladder epithelium, which leads to recrudescence (Anderson et al., 2003; Eto et al., 2007; Flores-Mireles et al., 2015).

UPEC achieves its adhesion primarily using filamentous adhesive structures like pili or fimbriae, and type 1 fimbriae, pyelonephritis associated pili (pap), and S fimbriae and are regulated by the *fim*, *pap*, and *sfa* operons, respectively. Furthermore, environmental cues can trigger expressional switches between pilus operons (phase variation) to increase the probability of adherence to host tissue (Snyder et al., 2005). PapG and FimH are the adhesin tips of p and type-1 fimbriae, respectively, that aid the development of cystitis after UPEC invasion (Hahn et al., 2002; Eto et al., 2007). Curli fimbriae are a type of amyloid fiber that facilitates surface adhesion, cell aggregation, and biofilm formation in the family *Enterobacteriaceae* (Kikuchi et al., 2005). Curli formation is regulated by the *csg*DEFG and *csg*AB operons (Chapman et al., 2002), and Curli plays an essential role in the colonization of the urinary tract, biofilm formation, and the persistence of UPEC post-interaction with antimicrobial peptides (Luna-Pineda et al., 2019). Iron acquisition is essential for UPEC survival in iron-deficient environments like the urinary tract, and UPEC expresses siderophores (small-molecule iron-chelators that scavenge the ferric ion) such as enterobactin, which can outcompete transferrin (a mammalian iron carrier with a K_d_ of ~10^−45^) and enable UPEC survival in the urinary tract (Goetz et al., 2002; O'Brien et al., 2016).

Although novel remedies like vaccines, probiotics, pilicides, curlicides, and D-mannose derived FimH antagonists are being studied, antibiotics remain an integral part of the therapeutic armamentarium, and their usage invariably results in antimicrobial resistance (Seo et al., 2021). Thus, novel drugs are required that can suppress the above-mentioned plethora of virulence factors.

Indole is an intra- and extra-cellular signaling molecule that is produced by the tryptophanase gene in the prokaryotic system including more than 85 bacterial species and eukaryotic system (Boya et al., 2021; Kumar et al., 2021). Indole has many functions; for example, it plays crucial roles in biofilm formation, signaling, plasmid stability, and drug resistance (Lee and Lee, 2010) and prevents *E. coli* cell division by modulating membrane potential (Chimerel et al., 2012). Indole also induces xenobiotic multidrug expulsion gene expression (Hirakawa et al., 2005), and indole derivative 3-indolylacetonitrile was reported to inhibit *E. coli* O157:H7 biofilm formation (Lee et al., 2011).

In this study, we screened 83 indole derivatives for their effects on the growth, biofilm formation, and virulence of UPEC. The effects of the three most potent derivatives, 4-chloroindole, 5-chloroindole, and 5-chloro 2-methyl indole, were subjected to further study to determine their effects on curli production, cell-surface hydrophobicity, and motility and on polymicrobial biofilms and other nosocomial pathogens, namely, *Staphylococcus aureus*, *Pseudomonas aeruginosa*, *Acinetobacter baumannii*, and *Candida albicans*. In addition, the effects of these three indoles on the expressions of virulence genes were studied, and *in silico* analysis was performed to establish quantitative structure–activity relationships (QSARs) of the indole moiety and the ADME (adsorption, distribution, metabolism, and elimination) parameters of the three selected chloroindoles.

## Materials and Methods

### Indole and Its Derivative

Indole and indole derivatives were purchased from Sigma-Aldrich (St. Louis, MO, United States) or Combi-Blocks (San-Diego, California, United States). DMSO (dimethyl sulfoxide) was used as the solvent, and 100 mg/ml stock solutions were prepared and stored at −20°C until required. 0.1% (vol/vol) DMSO was used as the negative control; at this concentration, DMSO did not affect bacterial growth or biofilm formation. Details of all the indole derivatives examined are provided in Supplementary Table S1.

### Bacterial Culture and Growth Conditions

All experiments were conducted at 37°C in nutrient broth (NB) unless mentioned otherwise. Uropathogenic *E. coli* O6:H1 strain CFT073 (ATCC 700928) was streaked from −80°C glycerol stocks on Luria-Bertani (LB) agar plates, and single colonies were cultured in NB overnight at 250 rpm and 37°C. In this study, overnight cultures refer to a growth period of 12 h with bacteria grown to stationary phase, and their dilution at 1:100 has approximately 10^6^ CFU/ml. The UPEC strain was a clinical isolate of a highly virulent strain isolated from the blood of a woman with acute pyelonephritis (Welch et al., 2002). All the experiments were performed using at least two independent cultures in triplicate.

### Screening of Indole Derivatives, MIC and Antibiofilm Assessments, and 3D Representations of Biofilms

Overnight cultures of the UPEC strain were reinoculated at 1:100 dilution and added to 96-well polystyrene plates (SPL Life Sciences, Korea) with or without the 83 indole derivatives at concentrations 20, 50, 75, 100, 200, or 400 μg/ml and grown statically for 24 h at 37°C. Absorbances were recorded at 620 nm, and minimum inhibitory concentrations (MICs) were defined as the lowest of the tested concentrations that prevented bacterial growth. To quantify biofilm formation, plates were washed gently three times with dH_2_O (so as not to disturb biofilms), 0.1% crystal violet was added to wells, and were incubated for 20 min. Plates were re-rinsed with dH_2_O and the crystal violet was extracted with 95% ethanol. Absorbances were measured at 570 nm using a Multiskan EX microplate reader (Thermo Fisher Scientific, Waltham, MA, United States).

The three selected indole derivatives (4-chloroindole, 5-chloroindole, and 5-chloro 2-methyl indole) were then tested for their abilities to disrupt mature UPEC biofilms. Two similar 96-well plates were prepared as described above, and UPEC was grown statically for 24 h at 37°C. Plate absorbance was measured at 570 nm in one plate and media in the other plate was gently removed and replaced with fresh media containing indole derivatives at 0, 50, 100, or 200 μg/ml. The plate was then incubated for 24 h at 37°C, and absorbances were measured at 570 nm. For 3D representations, UPEC biofilms produced in the presence or absence of selected indoles at concentrations of 50 or 75 μg/ml were grown in 12-well plates with glass coverslips inserted vertically in wells for 24 h at 37°C. Coverslips were then gently washed with dH_2_O twice, stained with 0.1% crystal violet for 20 min, and re-washed to remove residual dye. Biofilm images were taken using the iRiS™ Digital Cell imaging system (Logos Biosystems, Annandale, VA, United States). 3D images were constructed using Image J software.^1^

### Bacterial Growth Curves

The time- and concentration-dependent growths of UPEC were monitored. Overnight cultures of UPEC were reinoculated at 1:100 dilution in NB with or without indole derivatives at concentrations of 50 and 100 μg/ml in 250-ml flasks and incubated at 250 rpm and 37°C. Absorbances were measured at 600 nm using an Optizen 2120 UV spectrophotometer (Mecasys Co. Ltd., Daejeon) every 2 for 12 h and at the 24th hour.

### Rapid Killing Assay

Overnight cultures were reinoculated at 1:100 dilution with or without the three indole derivatives, tetracycline, kanamycin, gentamycin, or ciprofloxacin at concentrations 100 or 200 μg/ml and incubated at 250 rpm and 37°C. Cultures were then serially diluted using appropriate dilution factors (10^−4^, 10^−5^, or 10^−6^) and plated on LB agar plates at 30 or 60 min after incubation. Colony-forming units (CFUs) on the plates were measured after incubation for 24 h and at 37°C. CFU per ml is calculated as follows:


$$\begin{array}{l} \mathrm{CFU}/\mathrm{ml}=\mathrm{Number of colony units}\times \\ \;\;\;\;\;\;\;\;\;\;\;\;\;\;\;\;\;\;\;\;\;\;\;\;\;\;\;\;\;\mathrm{dilution factor}/\mathrm{volume plated}\;\left(\mathrm{ml}\right) \end{array}$$

### Scanning Electron Microscopy of UPEC Biofilms

Overnight cultures were inoculated at 1:100 dilution and were added to a 96-well polystyrene plate with or without indole derivatives at 50 μg/ml (as described in Section “Screening of Indole Derivatives, MIC and Antibiofilm Assessments, and 3D Representations of Biofilms”). Nitrocellulose membranes (3 × 3 mm) were also added to each well of the plate and incubated for 24 h. Membranes with adherent biofilms were then fixed using a 2.5% glutaraldehyde/2% formaldehyde solution and incubated overnight at 4°C, stained with OsO_4_, dehydrated using a graded ethanol series (30%, 50%, 70%, 90%, 95%, and 100%), placed in isoamyl acetate, critically point dried, and sputter-coated with gold. SEM was performed at 15 kV using a field emission-scanning electron microscope (FE-SEM; S-4200, Hitachi, Tokyo, Japan) at various magnifications.

### Swarming and Swimming Motilities

Swarming motility was assayed using 0.5% LB agar plates supplemented with 0.8% glucose (w/v), and swimming motility was assayed using 0.3% agar plates containing 1% tryptone (w/v) and 0.25% NaCl (w/v) with or without the three selected indole derivatives at 20, 50, or 100 μg/ml. Fresh colonies of uropathogenic *E. coli* CFT073 from LB agar plates were inoculated in 14-ml tubes containing 2 ml of LB medium and grown to an OD of 1.0 at 600 nm. Aliquots (0.2 μl) of these cultures were spotted on assay plates using sterilized micropipette tips and incubated for 24 h at 37°C. Average motility halo diameters were measured.

### Congo Red Agar Assay

Congo red agar assays were performed as previously described with some modifications (Shafreen et al., 2017). Mueller–Hilton agar plates supplemented with 0.04% Congo red (w/v) and 5% sucrose (w/v) with or without indole derivatives at 25 or 50 μg/ml were spotted with 20 μl of overnight UPEC culture and incubated for 48 h at 37°C.

### Indole Assay

Indole concentrations were determined using Kovac’s reagent as previously described (Darkoh et al., 2015). Overnight UPEC cultures were re-inoculated at 1:100 dilution with or without indole derivatives at concentrations of 25 or 50 μg/ml and incubated with shaking at 250 rpm for 10 h at 37°C. Cultures were centrifuged at 10,950×*g* for 10 min and 300 μl Kovac’s reagent (0.05% (w/v) of *p*-dimethyl amino benzaldehyde dissolved in a 1:3 ratio of HCl and amyl alcohol) was added to the supernatant and incubated for 2 min. Aliquots (50 μl) of the pink-colored suspensions on the tops of mixtures were added to 1 ml of HCl-amyl alcohol solution (1:3 ratio), and absorbances were measured at 540 nm.

### Reactive Oxygen Species Assay

Briefly, UPEC cells grown overnight were harvested in NB, resuspended at 10^6^ CFU/ml in PBS, treated with or without H_2_O_2_ (positive control) or indole derivatives at 25 or 50 μg/ml for 1 h at 250 rpm and 37°C, and then, 2′,7′-dichlorofluorescein diacetate (5 μM) was added to cell suspensions and incubated in the dark for 30 min at 37°C. Fluorescence was measured using a multimode microplate reader JASCO-F-2700 (Hitachi, Tokyo, Japan) equipped with a xenon arc lamp. Excitation and emission slits were fixed at 5 nm, the excitation wavelength was at 506 nm, and emission intensities were recorded at 524 nm. Fluorescence intensities (FI)/OD_600_ were normalized for growth. Untreated cells were processed similarly and used as controls. UV–Vis spectra of samples were obtained using a 3220 UV spectrometer (Optizen, Daejeon, South Korea) using quartz cuvettes (1 cm path length) from 200 to 800 nm at a wavelength uncertainty of ±2 nm.

### Cell Surface Hydrophobicity

Cell surface hydrophobicity was quantified as previously described (Rosenberg et al., 1980). UPEC cultures were prepared from overnight cultures at 1:100 dilution with or without the selected three indole derivatives at 25 or 50 μg/ml and incubated with shaking at 250 rpm for 24 h at 37°C. Cell suspensions of 1 ml were then centrifuged at 15,300×*g* for 10 min, and the pellets obtained were washed twice with PBS and resuspended in 1 ml of PBS. Xylene (250 μl) was added to PBS buffered cell suspensions, vortexed vigorously, and left undisturbed for 30 min. The 1 ml PBS blank was processed similarly. The OD values before vortexing (A_0_) and absorbances of aqueous phases (A_i_) were measured at 600 nm_._ Percent hydrophobicities are calculated using the following formula:


$$Percenthydrophobicity\;\left(\%H\right)=\left(A_{0}-A_{i}\right)\times 100/A_{i}$$

### RNA Extraction and qRT-PCR

Twenty-five milliliters of NB was inoculated with overnight cultures of UPEC at 1:100 dilution, incubated at 250 rpm and 37°C, and grown to an OD_600_ of 1.0. Cultures were then incubated with or without the three selected indole derivatives at 100 μg/ml at 250 rpm for 3 h at 37°C. RNase inhibitor (RNAlater, Ambion, TX, United States) was then added to prevent RNA degradation. Cells were harvested centrifuging cultures at 15,300×*g* for 5 min at 4°C. RNA was isolated using RNeasy Mini Kits (Valencia, CA, United States), and RNA concentration (ng/μl) and purity (A_260_/A_280_ ratio) were measured using a NanoVue Plus nanodrop spectrophotometer (GE, Chicago, IL, United States). qRT-PCR was used to check the expressions of biofilm, virulence, and motility-related gene expressions. The primers used and their sequences and functions are provided in Supplementary Table S2. The *rrsG* gene was used as an endogenous control. PCR was performed using SYBR Green master mix and an ABI StepOne Real-Time PCR system (Applied Biosystems, Foster City, CA, United States). Four reactions per gene were performed using two independent cultures.

### *In silico* Analysis: 3D QSAR and ADME Properties

3D QSAR model was developed as previously described with some modifications (Varpe et al., 2020). The 3D structures of 73 of the 83 indole derivatives were downloaded from PubChem, and macromolecule energy minimization was performed using the Ligprep module and the OPLS2005 force field. Substructure alignments of indole derivatives to indole were performed in Maestro 12.5 (Schrodinger Software solutions, United States). The MICs of derivatives were converted to pMIC [−log (MIC)] values, and a 3D atom-based QSAR model was built using PHASE (Schrodinger Software solutions, United States) with 70:30 randomization into the training and test sets. Five PLS (partial least square) factor models were built, and the 5th model was chosen for QSAR visualization and activity predictions. External validation of the model was done using the remaining 10 indole derivatives with predetermined MICs. The QSAR PLS factor models and their activity predictions and external validations are provided in Supplementary Tables S3–S5.

ADME, drug-likeness, and toxicity profiles of the indole derivatives were evaluated using online webservers, *viz.* PreADMET,^2^ Molinspiration,^3^ and GUSAR (all accessed on 8 September 2021).^4^ Property validation was performed using server bioassay parameters or cited references. A summary of the ADME properties of the indole derivatives is provided in Supplementary Table S6.

### Antimicrobial and Antibiofilm Activities of Indole Derivatives Against Other Nosocomial Pathogens

MICs and antibiofilm activities of the three selected indole derivatives against other nosocomial pathogens, that is, *S. aureus* ATCC 6538, *P. aeruginosa* PAO1, *A. baumannii* ATCC 17978, and *C. albicans* DAY 185, were studied using the methods described in section “Screening of Indole Derivatives, MIC and Antibiofilm Assessments, and 3D Representations of Biofilms.” LB was used for *S. aureus* and *P. aeruginosa*, whereas tryptic soy broth (TSB) and potato dextrose broth (PDB) were used for *A. baumannii* and *C. albicans*, respectively, in the presence or absence of indole derivatives at concentrations 20, 50, or 100 μg/ml at 250 rpm and 37°C. The re-inoculation of *C. albicans* overnight cultures into PDB was performed at a dilution of 1:50, while others were done at a dilution of 1:100 in their respective media. The cultures were incubated at 37°C for 24 h.

### Antibiofilm Activities of Indole Derivatives Against Polymicrobial Biofilms

The antibiofilm activities of indole derivatives against polymicrobial *S. aureus* ATCC 6538, *C. albicans* DAY 185, and UPEC CFT073 biofilms were performed as previously as described in section “Screening of Indole Derivatives, MIC and Antibiofilm Assessments, and 3D Representations of Biofilms.” Equal parts of LB, NB, and PDB with or without indole derivatives at concentrations of 20, 50, or 100 μg/ml were used as media for polymicrobial biofilm formation (final volume per well = 300 μl). The cultures were incubated at 37°C for 24 h.

### Confocal Laser Scanning Microscopy of Polymicrobial Biofilms

Polymicrobial biofilms were formed in 96-well polystyrene plates as described in section “Antibiofilm Activities of Indole Derivatives Against Polymicrobial Biofilms” with or without indole derivatives at a concentration of 100 μg/ml. To visualize biofilms, wells were stained with carboxyfluorescein diacetate succinimidyl ester (Invitrogen, Molecular Probes, Inc., Eugene, United States). Biofilm structures were evaluated by visualizing plate bases using a 488-nm excitation Ar laser (emission 500–550 nm) under a confocal laser microscope (Nikon Eclipse Ti, Tokyo, Japan), and their spatial characteristics were quantified using the MATLAB R2007b program by analyzing at least four random positions in three independent cultures. To measure biofilm formation, color confocal images (20 image stacks) were converted to greyscale using ImageJ. MATLAB R2007b biofilm software was used to determine biomasses (μm^3^ per μm^2^), mean thicknesses (μm), and substratum coverages (%).

### Scanning Electron Microscopy of Polymicrobial Biofilms

Polymicrobial biofilms were formed in 96-well polystyrene plates containing a nitrocellulose membrane (3×3 mm) in each well, as described in section “Antibiofilm Activities of Indole Derivatives Against Polymicrobial Biofilms,” with or without indole derivatives at a concentration of 100 μg/ml. Membrane processing and SEM analysis were performed as described in section “Scanning Electron Microscopy of UPEC Biofilms.”

### Statistical Analysis

All experiments were performed using at least two independent bacterial cultures in triplicate. Data represent means ± SD. Statistical validation was performed using the Student’s *t* test under the assumption of a null hypothesis, and *p* ≤ 0.05 were considered as significant. Graphs were plotted using Sigma Plot Ver.14.0.

## Results

### Screening of Indole Derivatives for Antibacterial and Antibiofilm Activities

The 83 indole derivatives, which possessed various functional groups (like halogen, methyl, benzoyl, methoxy, nitro, formyl, or carboxylic acid) were screened for their antimicrobial and antibiofilm properties against UPEC. Screening was performed using crystal violet biofilm assays and by determining MICs (Supplementary Table S1). Of the 83 indole derivatives, three chloroindoles—4-chloroindole (4CI), 5-chloroindole (5CI), and 5-chloro 2-methyl indole (5CMI) demonstrated the best MIC/biofilm inhibition combination with MICs of 75 μg/ml and with biofilm inhibition percentages of 72, 66, and 64, respectively, at 20 μg/ml (Supplementary Table S1) and were selected for further analysis of their antivirulence properties against UPEC.

**Figure 1:**
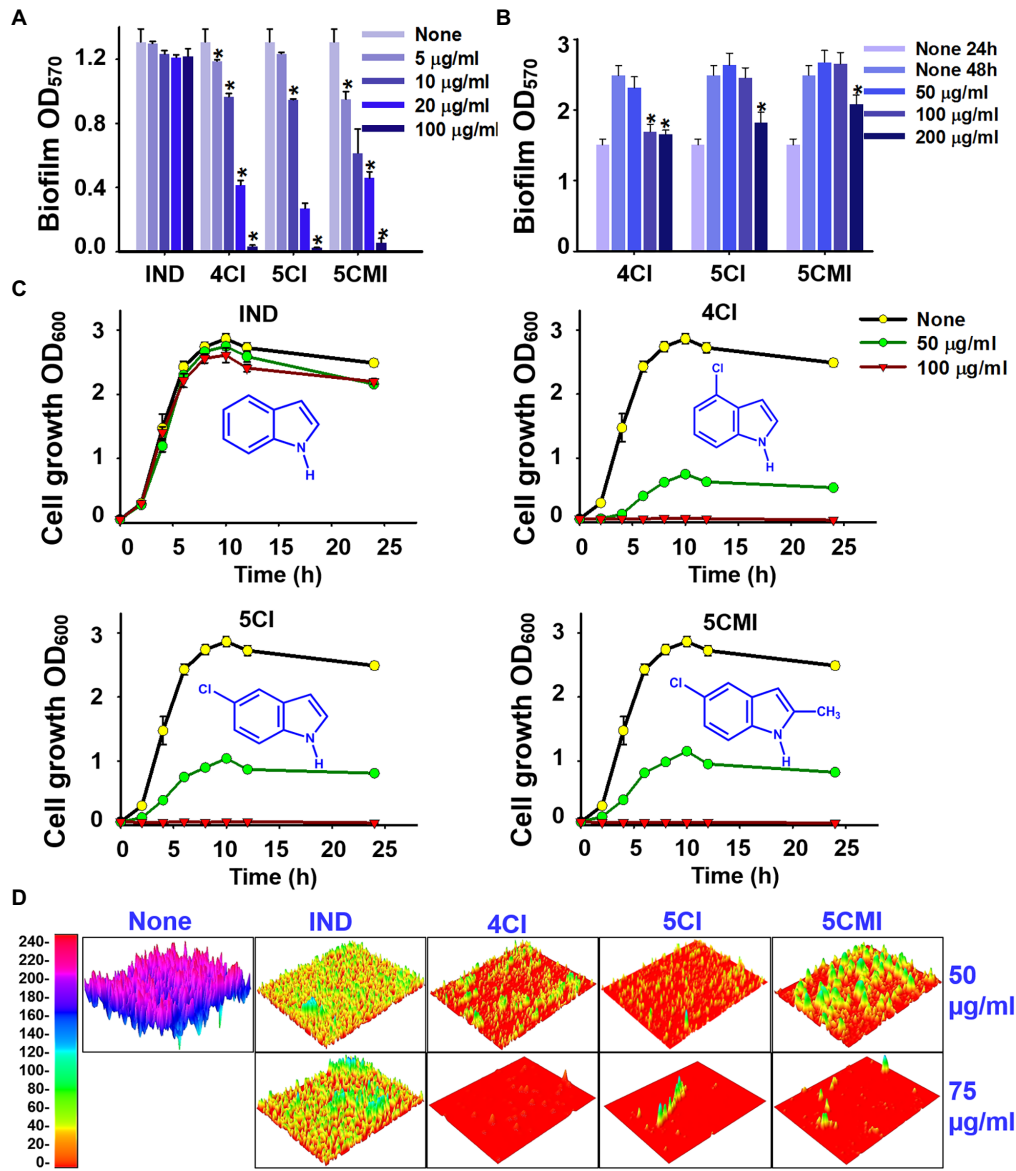
**(A)** Crystal violet quantification of biofilm formation in UPEC in the presence of indoles, wherein biofilm OD_570_ represents absorbance of CV-stained biofilm, **(B)** crystal violet biofilm dispersal assessment of UPEC in the presence of indoles, wherein biofilm OD_570_ represents absorbance of CV-stained biofilm, **(C)** cell growth curves of UPEC in the presence of indoles, and **(D)** 3D representation of UPEC biofilms in the presence of select indoles at concentrations 50 and 75 μg/ml. *p < 0.05 vs. non-treated controls.

4CI, 5CI, and 5CMI dose-dependently inhibited biofilm formation at concentrations of 5, 10, 20, and 100 μg/ml (Figure 1A). In contrast, indole did not inhibit biofilm formation (Figure 1A). Interestingly, these three chloroindoles did not disrupt pre-formed biofilms even at 200 μg/ml (Figure 1B), but reduced biofilm formation significantly at 24–48 h after biofilm maturation at a concentration of 200 μg/ml (Figure 1B). Hence, the ability of biofilm dispersal was weak with these three indoles.

UPEC cell growth was monitored in the presence of indole and each of the three chloroindoles. Indole did not affect the growth of UPEC at 50 or 100 μg/ml (Figure 1C), but 4CI, 5CI, and 5CMI dose-dependently inhibited cell growth (Figure 1C). At 100 μg/ml, 4CI, 5CI, and 5CMI completely inhibited UPEC cell growth (Figure 1C). The 3D representation of UPEC biofilms in Figure 1D shows that these three indoles markedly inhibited biofilm formation.

A rapid killing activity assay was also performed to compare the antimicrobial abilities of 4CI, 5CI, and 5CMI against some common antibiotics. 4CI, kanamycin, and gentamycin most potently reduced CFU counts from 5 × 10^6^ to 10 CFU/ml in 1 h at 200 μg/ml (Supplementary Figure S1). 5CI, 5CMI, ciprofloxacin, and tetracycline reduced CFU counts to 1*10^6^ CFU/ml in 1 h at 200 μg/ml (Supplementary Figure S1).

**Figure 2:**
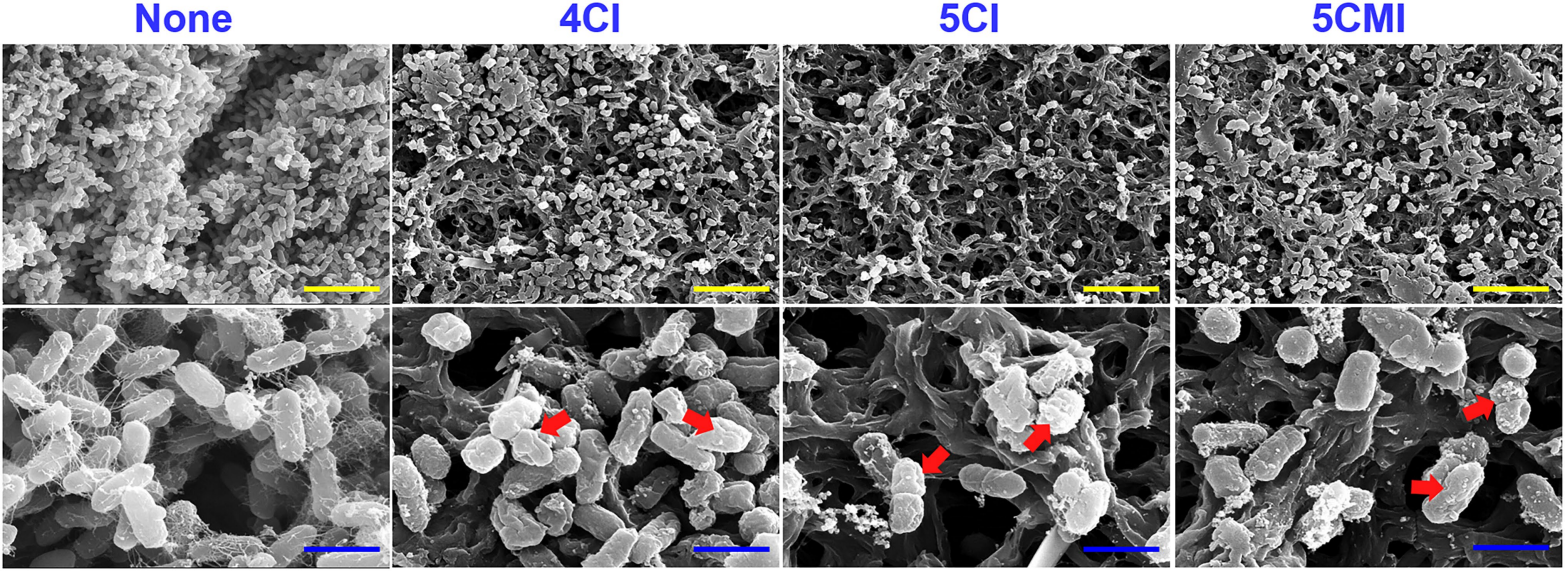
Scanning electron microscopy of UPEC biofilms in the presence of indoles (50 μg/ml) on nitrocellulose membranes. The red arrow represents cell membrane damage and shrinkage visualized at ×15,000 magnification. Yellow and blue scale bars represent 10 and 2 μm, respectively.

Scanning electron microscopy of UPEC biofilms on nitrocellulose membranes showed inhibition of UPEC biofilm formation by the three chloroindoles at 50 μg/ml (Figure 2). Visual inspection showed cell viabilities were also reduced (Figure 2). At high magnification (×15,000), cells in treated samples appeared deformed and shrunken.

### Inhibition of Swarming and Swimming Motilities by Indoles

**Figure 3:**
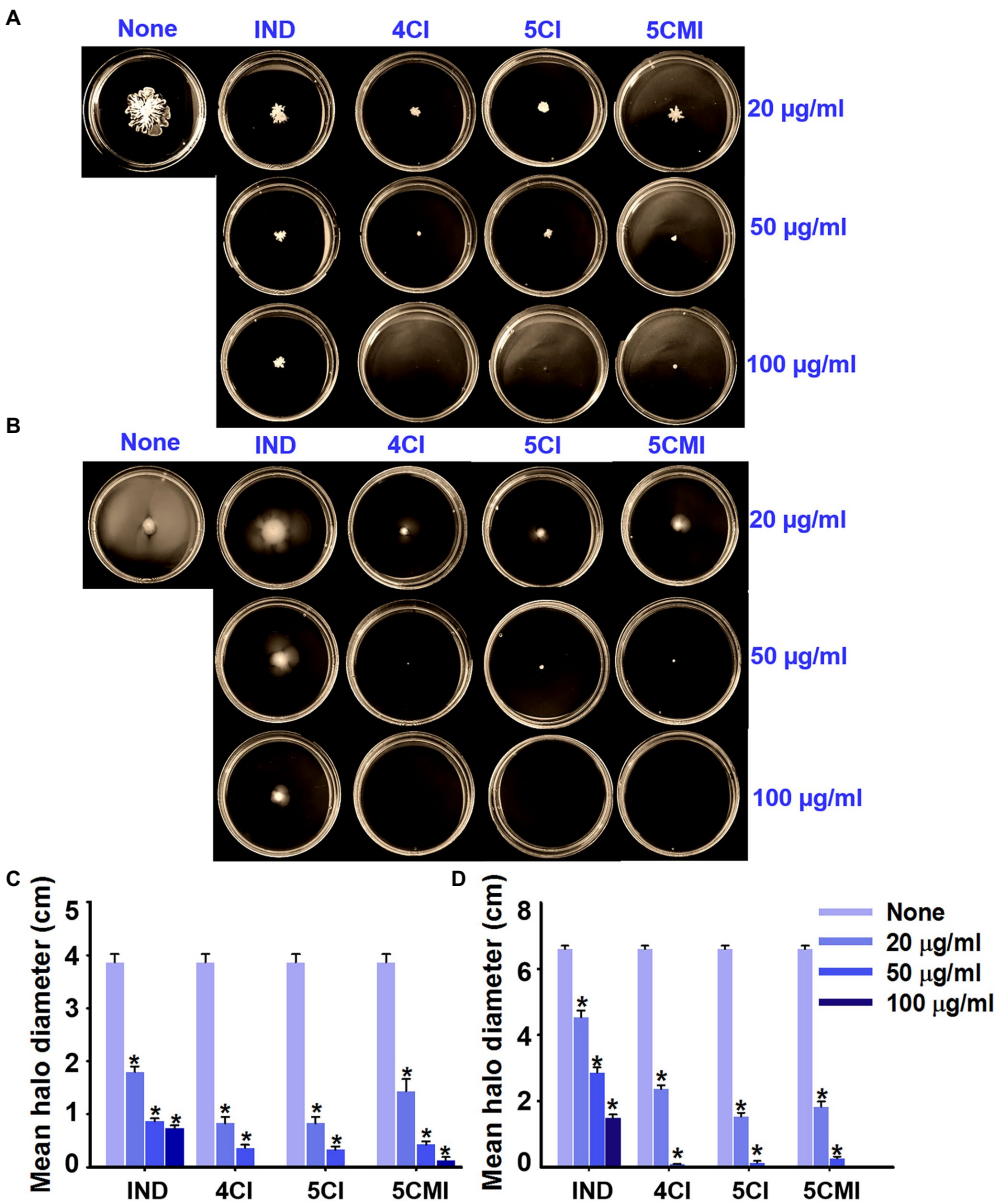
**(A)** Swarming and **(B)** swimming motility assay results for UPEC in the presence of indole, 4CI, 5CI, or 5CMI. Mean **(C)** swarming and **(D)** swimming motility halo diameters of indole and the three derivatives. *p < 0.05 vs. non-treated controls.

Swarming and swimming motilities of UPEC were assayed in the presence of 4CI, 5CI, or 5CMI. All three inhibited swarming and swimming motilities (Figures 3A,B). Mean swarming and swimming motility halo diameters without treatment were 3.9 and 6.6 cm, respectively (Figures 3C,D). Even at a low concentration of 20 μg/ml, all three indole derivatives inhibited motility by ~65% inhibition vs. non-treated controls (Figures 3C,D). At the concentration of 100 μg/ml, swarming and swimming motilities were completely abolished by the chloroindoles (Figures 3C,D). Although indole also dose-dependently inhibited motilities, its effects were not as pronounced as those of the three derivatives (Figures 3C,D).

### Inhibition of Curli Production by Indoles

**Figure 4:**
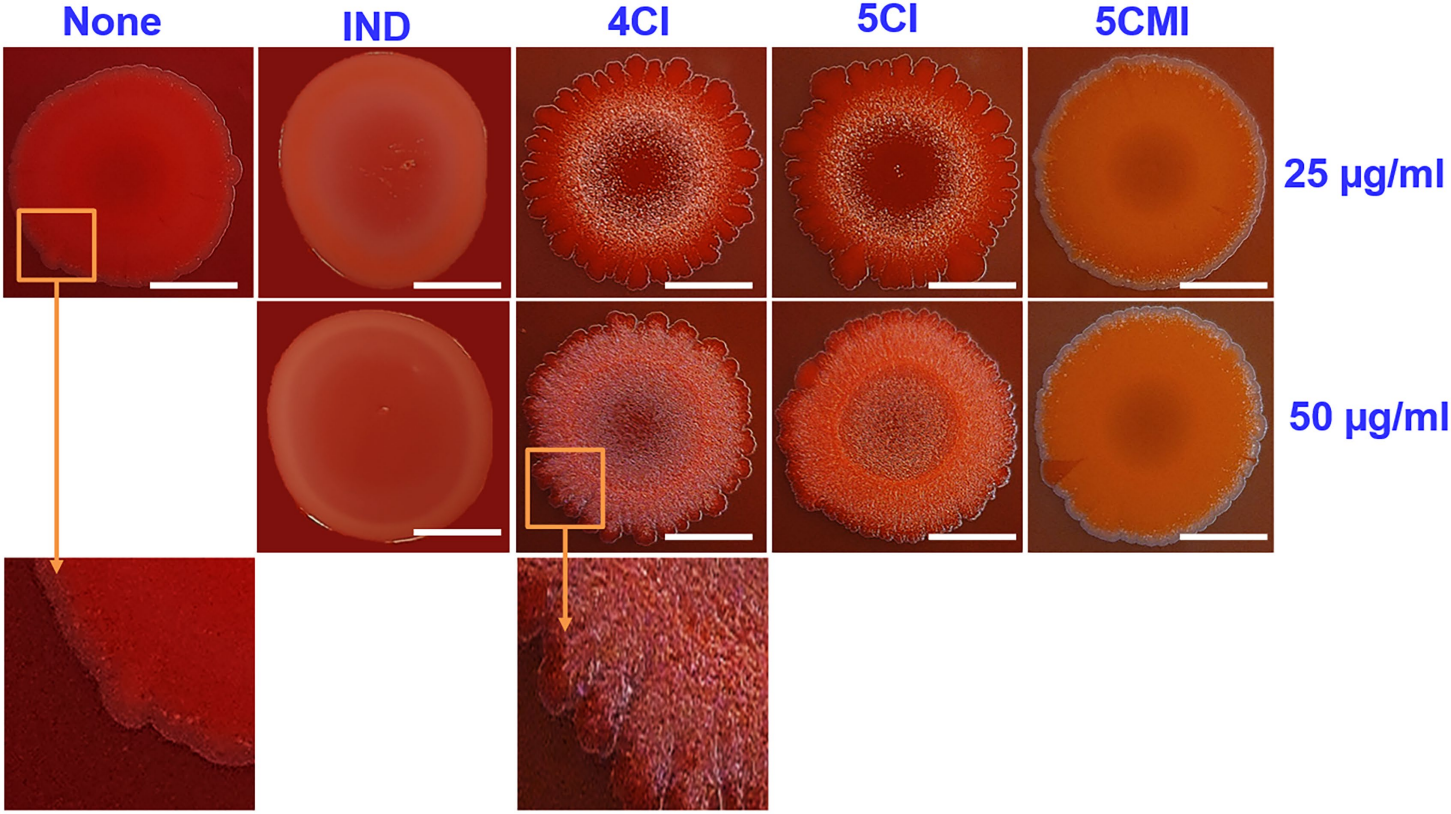
Congo red curli assay of UPEC in the presence of indoles at concentrations of 25 and 50 μg/ml. White scale bars represent 1 cm.

A Congo red agar assay was used to determine the effects of the select indole derivatives on curli production. The inoculum morphology observed concurred with that previously reported (Shafreen et al., 2017), wherein a clinical isolate of *E.coli* exhibited curli binding with Congo red agar. Untreated samples showed a red, dry, and rough morphology indicating binding to Congo red agar (Figure 4), whereas treatment with the select indole derivatives resulted in a pale and wrinkled morphology, indicating less binding. The wrinkle zone of the inoculum in treated samples also broadened dose-dependently. Interestingly, indole produced a pale inoculum but did not cause wrinkling (Figure 4).

### Effects of Indoles on Production of Extracellular Indole, Reactive Oxygen Species, and Cell Surface Hydrophobicity

**Figure 5:**
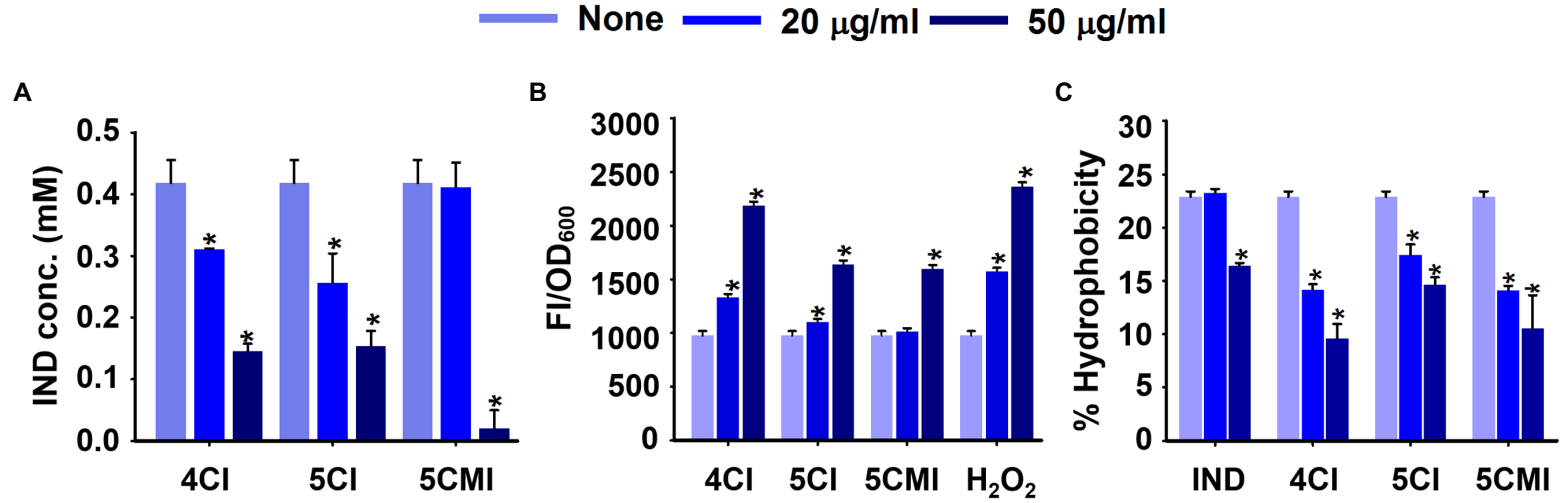
**(A)** Extracellular indole concentrations of UPEC in the presence of indole derivatives, **(B)** intracellular ROS production levels in UPEC in the presence of indoles or the positive control H_2_O_2_, and **(C)** Cell surface hydrophobicities of UPEC in the presence of indoles. *p < 0.05 vs. non-treated controls.

Indole assay was used to measure extracellular indole concentrations after incubation for 10 h with 4CI, 5CI, or 5CMI (Figure 5A). All three derivatives reduced extracellular indole concentrations significantly at 50 μg/ml (Figure 5A) post-normalization with cell growth. Extracellular indole concentrations were 0.14, 0.15, and 0.02 mM, respectively, after treatment with 4CI, 5CI, or 5CMI at 50 μg/ml, which were significantly lower than the 0.42 mM observed for the control.

Reactive Oxygen Species (ROS) assay results showed 4CI, 5CI, and 5CMI at 50 μg/ml significantly increased fluorescence intensities, indicating an increase in ROS concentration (Figure 5B). 4CI, 5CI, and 5CMI at 50 μg/ml increased ROS production by 2.2-, 1.7-, and 1.6-fold, respectively (Figure 5B). H_2_O_2_ at 50 μg/ml increased ROS production 2.4-fold.

We also assessed UPEC cell surface hydrophobicities after treatments with the three derivatives (Figure 5C). 4CI, 5CI, and 5CMI significantly inhibited cell surface hydrophobicity at 20 and 50 μg/ml (Figure 5C). At 50 μg/ml, 4CI, 5CI, and 5CMI reduced cell surface hydrophobicity by 13%, 8%, and 12%, respectively. Interestingly, indole inhibited hydrophobicity only at the concentration of 50 μg/ml (Figure 5C).

### Gene Expressions in UPEC After Treatment With the Chloroindoles

**Figure 6:**
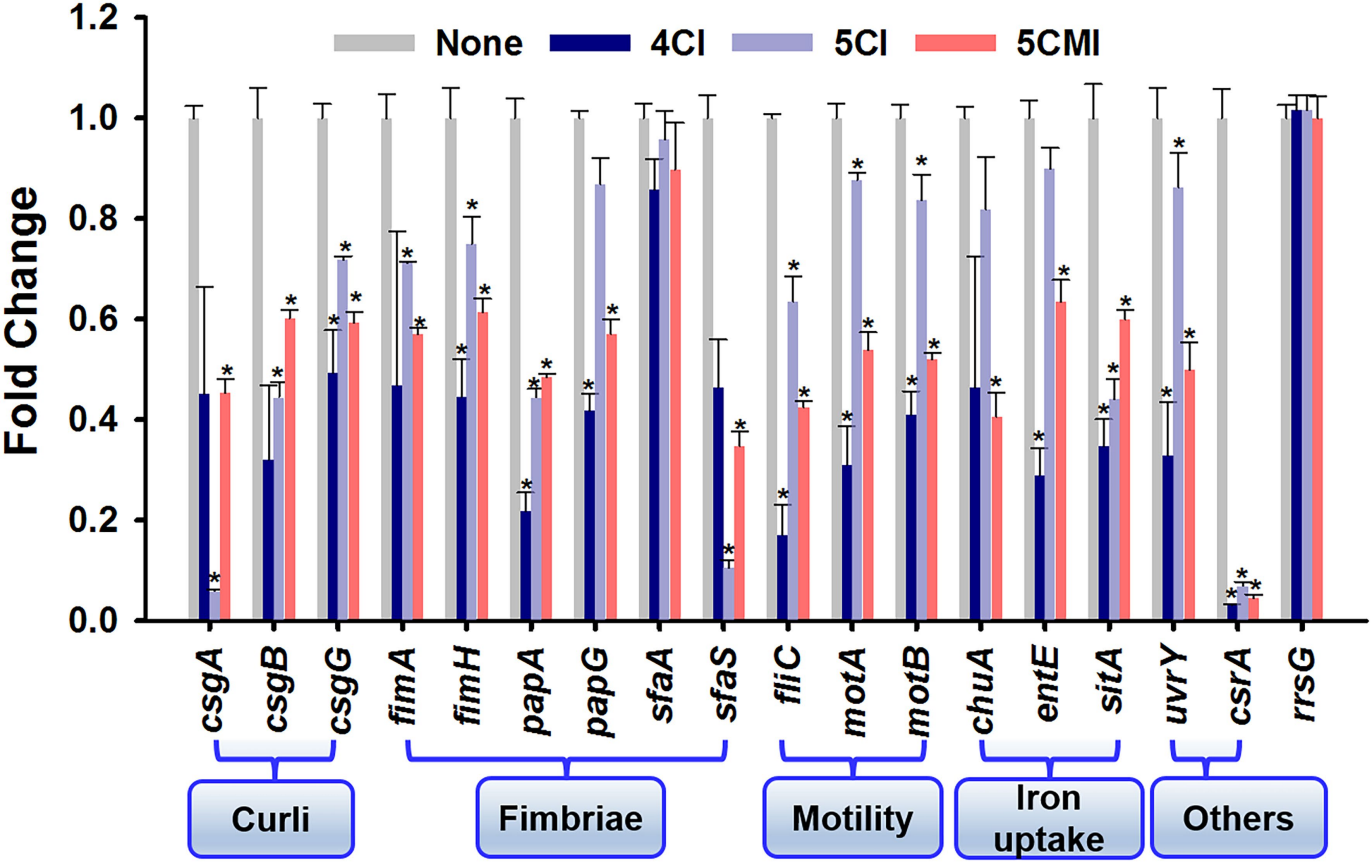
Gene expression profiles in the presence of indoles. *p < 0.05 vs. non-treated controls. The *rrsG* gene was used as a housekeeping control.

The effects of 4CI, 5CI, and 5CMI on the expressions of 17 virulence and biofilm-related genes (motility, curli, fimbriae, flagella, iron acquisition, and stress regulation) were examined (Figure 6). Overall, treatments downregulated the expression profiles of these genes, and the three chloroindoles exhibited similar downregulation patterns, though 4CI had more significant effects than 5CI or 5CMI. The 16 s rRNA (*rrsG*) sequence was used as the endogenous control, and its expression was unaffected by treatments. 4CI, 5CI, and 5CMI significantly downregulated the triad of curli genes (*csgA*, *csgB*, and *csgG*) by 3.0-, 2.4-, and 1.7-fold, respectively. The *fimA* and *fimH* genes were significantly downregulated by the three chloroindoles by an average 2.0- and 1.5-fold, respectively. Motility is mainly achieved by flagella, and *fliC*, which encodes flagellin subunits, was downregulated by an average of 3.5-fold by the three derivatives, which was substantially greater than their effects on the chemotaxis genes *motA* and *motB* (downregulated by 2.0- and 1.8-fold, respectively). P fimbriae and S fimbriae genes *papA*, *papG*, and *sfaS* were also significantly downregulated by the three derivatives by 2.9-, 1.9-, and 4.6-fold, respectively, on average. Interestingly, none of the three derivatives significantly downregulated the *sfaA* gene. Heme uptake *via* ChuA protein coded by the *chuA* gene is important for UPEC dissemination to bladder and kidneys and was also downregulated by the three derivatives by an average of 1.9-fold. Iron acquisition is performed by siderophores like enterobactin and improves survival in the urinary tract, and its encoding gene *entE* was downregulated on average by 2.1-fold (Henderson et al., 2009). Because the indole derivatives demonstrated antimicrobial effects and caused the shrinkage and damage of UPEC membranes (Figure 2), we checked their effects on the *sitA* gene, which is responsible for iron and manganese transport and oxidative stress regulation (Sabri et al., 2006). The selected indole derivatives were found to downregulate the *sitA* gene significantly by an average of 2.3-fold and also to significantly downregulate the two-component system *uvrY* gene and the global carbon storage regulator *csrA* gene by 2.2- and 18.5-fold, respectively.

### 3D-QSAR and ADME Profiling of Indole Derivatives

**Figure 7:**
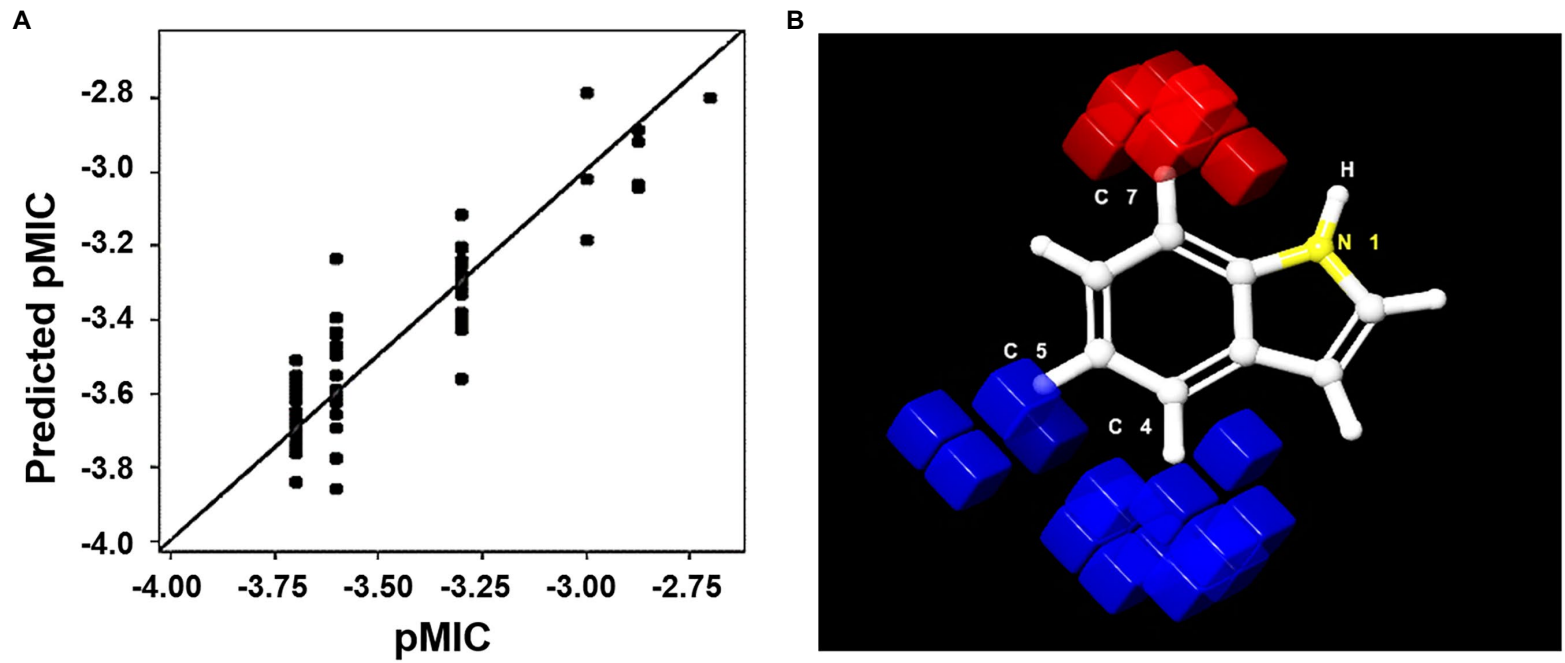
**(A)** Scatter plot of pMIC vs. predicted pMIC for the 73 indole derivatives, predicted by 3D-QSAR PLS factor 5 model. **(B)** 3D representation of positive (blue) and negative (red) positions of the indole moiety for MIC activity.

An atom-based QSAR model was developed using 73 of the 83 indole derivatives; the remaining 10 derivatives were used for external validation. Of the developed models, we chose to use the PLS factor 5 model, which had an *R*^2^ value of 0.9033, a stability of 0.809, an *F* value of 89.7, and a low standard deviation and RMSE (0.093 and 0.21, respectively). The predicted model predicted the activities of the predetermined MICs of the 73 indole derivatives (Figure 7A; Supplementary Table S4). External validation was performed to determine whether the model accurately predicted the MICs of the remaining 10 derivatives (Supplementary Table S5). The predicted model was used to identify regions of the indole moiety that might be helpful in future ligand-based drug screenings. We found that substitutions at the fourth and fifth indole positions favored antimicrobial activity, whereas the seventh position had unfavorable effects. These favorable and unfavorable effects are represented by blue and red cubes, respectively, in Figure 7B. Contours of these cubes were determined using various properties like the presence of a hydrogen bond donor and acceptor, hydrophobicity, and compatibilities of ionic features with receptor.

ADME profiles (adsorption, distribution, metabolism, and excretion) of the three selected indole derivatives were examined. 4CI, 5CI, and 5CMI did not violate Lipinski’s rule of five, had excellent skin and human intestinal adsorptions, good miLogP values, did not exhibit acute fish or algal toxicity, and were also non-carcinogenic to mice. The full ADME profile is presented in Supplementary Table S6.

### Effects of the Three Chloroindoles on Other Nosocomial Pathogens and Polymicrobial Biofilms

**Figure 8:**
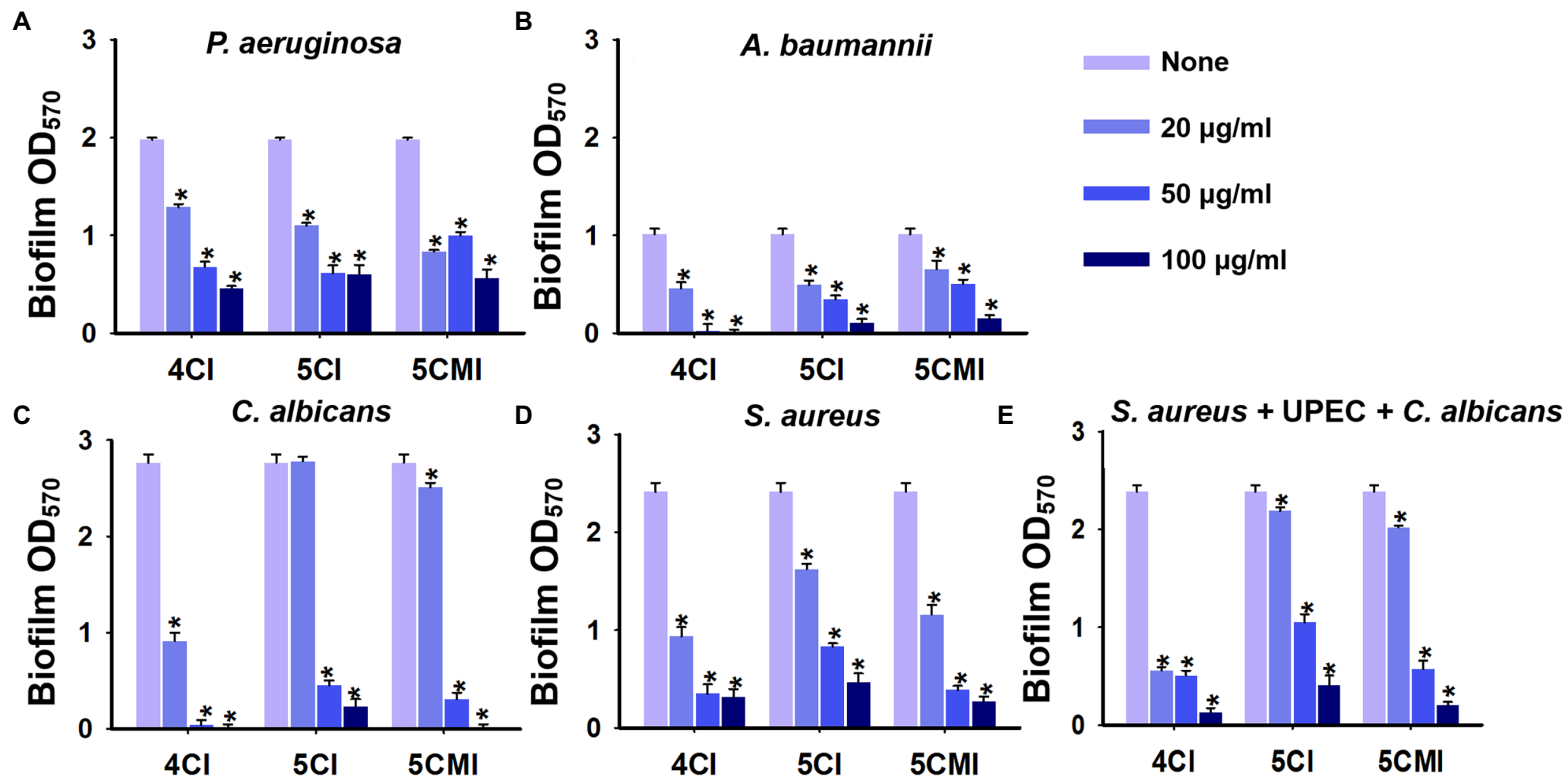
Crystal violet quantification of biofilm inhibition by the three chloroindoles of **(A)** *Pseudomonas aeruginosa* PAO1, **(B)** *Acinetobacter baumannii* ATCC 17978, **(C)** *Candida albicans* DAY 185, **(D)** *Staphylococcus aureus* ATCC 6538, and **(E)** a polymicrobial biofilm (*S. aureus* ATCC 6538, *C. albicans* DAY 185 and UPEC) grown in media comprised of equal parts of Luria-Bertani (LB), nutrient broth (NB), and potato dextrose broth (PDB) broth. Chloroindoles concentrations used are 0, 20, 50, and 100 μg/ml. The incubation time for all the conditions is 24 h at 37°C. *p < 0.05 vs. non-treated controls.

4CI, 5CI, and 5CMI also significantly inhibited biofilms formed by other nosocomial pathogens. Biofilm formation confers *P. aeruginosa* with antimicrobial resistance, and this leads to UTIs and possibly cystic fibrosis (Drenkard, 2003). Chloroindoles dose-dependently inhibited biofilm formation and at 100 μg/ml inhibited biofilm formation by *P. aeruginosa* PAO1 by 78% (Figure 8A). *Acinetobacter baumannii* is a nosocomial pathogen that causes infections of the urinary tract and bloodstream, osteomyelitis, and meningitis (Peleg et al., 2008), and the chloroindoles dose-dependently inhibited *A. baumannii* ATCC 17978 biofilm formation and at 100 μg/ml inhibited its biofilm formation by 100% (Figure 8B). On the other hand, *C. albicans* is a nosocomial fungal pathogen that can cause candidiasis and form biofilms on medical devices (Pappas et al., 2004). 4CI, 5CI, and 5CMI dose-dependently inhibited biofilm formation by *C. albicans* DAY 185 and at 100 μg/ml completely inhibited its ability to produce biofilms (Figure 8C). Chloroindoles also dose-dependently inhibited biofilm formation by *S. aureus* ATCC 6538 and at concentration of 100 μg/ml inhibited biofilm formation by 88% (Figure 8D). *Staphylococcus aureus* is a commensal bacterium responsible for several types of systemic infections like bacteremia, infective endocarditis, and other skin and soft tissue infections (Tong et al., 2015). It can also produce multispecies biofilms with *C. albicans* on medical implants (Schlecht et al., 2015). The three selected chloroindoles also had antimicrobial activities against the above-mentioned nosocomial pathogens except *P. aeruginosa* PAO1. 4CI possessed the most potent antimicrobial activity with a MIC of 50 μg/ml against *S. aureus*, *A. baumannii*, and *C. albicans*. Both 5CI and 5CMI had a MIC of 100 μg/ml against *S. aureus*, *A. baumannii*, and *C. albicans* except 5CI for *A. baumannii*, wherein the MIC was 50 μg/ml. The MICs of the three chloroindoles for nosocomial pathogens are shown in Supplementary Table S7.

**Figure 9:**
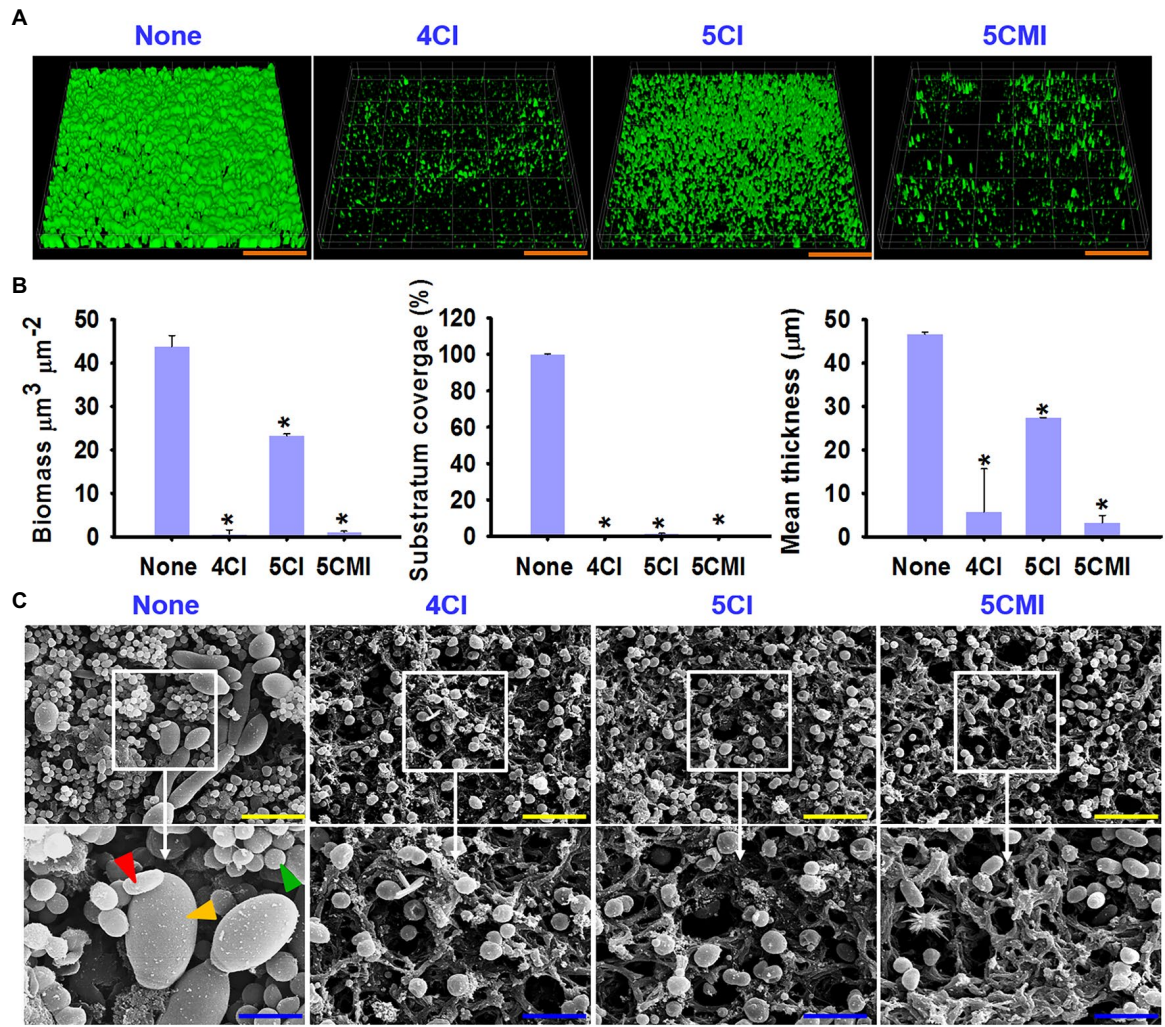
**(A)** Confocal laser scanning microscopy (CLSM) images of polymicrobial biofilms (*S. aureus* ATCC 6538, *C. albicans* DAY 185, and UPEC) grown in the presence of the three indole derivatives at 100 μg/ml, **(B)** COMSTAT analysis of polymicrobial biofilms (*S. aureus* ATCC 6538, *C. albicans* DAY 185, and UPEC), **(C)** SEM images of polymicrobial biofilms (*S. aureus*, UPEC, and *C. albicans*) grown in the presence of the three indole derivatives at 100 μg/ml. Red, yellow, and green arrows represent UPEC, *C. albicans*, and *S. aureus*, respectively. *p < 0.05 vs. non-treated controls. Orange, yellow, and blue scale bars represent 100, 10, and 3 μm, respectively.

Triple species biofilms containing uropathogenic *E. coli* CFT073, *S. aureus* ATCC 6538, and *C. albicans* DAY 185, as previously described, were used as a model to determine the potencies of the three indole derivatives against polymicrobial biofilms (Kim et al., 2021). In our study, *S. aureus* is the most dominant of the three species used and outcompetes *C. albicans* and UPEC in biofilm formation as observed in other studies (Stoodley et al., 2012; Alves et al., 2018). 4CI, 5CI, and 5CMI significantly inhibited polymicrobial biofilm formation at concentrations of 50 and 100 μg/ml (Figure 8E). Confocal laser scanning microscopy (CLSM) of polymicrobial biofilms grown in the presence of these indole derivatives at 100 μg/ml showed significant reductions in biomass, substratum coverage, and mean thickness (Figures 9A,B). SEM analysis of polymicrobial biofilms provided further insight into biofilm inhibition and showed 4CI, 5CI, and 5CMI disrupted polymicrobial biofilm formation on nitrocellulose membranes (Figure 9C).

## Discussion

Indole derivatives constitute an important class of therapeutic agents with anticancer, antioxidant, antirheumatoidal, and antimicrobial activities (Kumari and Singh, 2019). In this study, we examined 83 indole derivatives for their antibiofilm and antimicrobial activities against UPEC. Of the 83 indole derivatives preliminarily screened, 4-iodoindole (4II), 5-iodoindole (5II), 6-iodoindole (6II), 4-chloroindole (4CI), 5-chloroindole (5CI), 5-chloro 2-methyl indole (5CMI), 4-bromoindole (4BI), and 5-bromoindole (5BI) had MICs of <100 μg/ml (Supplementary Table S1). Interestingly, halogenated indole derivatives were the most potent at primary screening. These indoles have been studied for their antimicrobial and antibiofilm activities on diverse microorganisms. It has been reported that halogenated indoles effectively reduced biofilm and hyphal formation by fungi like *C. albicans* and eradicated *B. cinerea* fruit infection (Manoharan et al., 2018; Raorane et al., 2022), reduced biofilm formation, virulence, and root surface colonization by *Agrobacterium tumefaciens* (Ahmed et al., 2022). In addition, they effectively killed metabolically dormant persister cells in *S. aureus* and *P. aeruginosa* biofilms (Manoharan et al., 2018). Although bromoindoles were the most potent derivatives in terms of inhibiting biofilm formation in the current study, ADME profiling of bromoindoles using the *pre-admet* server (results not shown) returned positive results for rat carcinogenicity and zebrafish embryo toxicity (Kammann et al., 2006; EC_50_ in the range of 4.3–9.2 μg/ml^30^), while 4CI, 5CI, and 5CMI did not show any toxicity (Supplementary Table S6).

Chloroindoles dose-dependently inhibited biofilm formation by UPEC but had little effect on pre-formed biofilms (Figures 1A,B), which showed the three selected chloroindoles inhibited rather than eradicated biofilms. Subsequent testing showed they rapidly killed bacteria at higher concentrations and had potencies similar to those of commonly prescribed antibiotics (Supplementary Figure S1). SEM showed all three inhibited EPS (extracellular polymeric substance) production (Figure 2), an essential component of biofilms. We suppose that the membrane damage and shrinkage of UPEC cells observed were the result of increased ROS levels (Figure 2). Furthermore, this observation concurs with a previous report that 5-iodoindole induced membrane damage and shrinkage in *E. coli* O157:H7 (Raorane et al., 2020). ROS production by UPEC cells was significantly increased by chloroindole treatments (Figure 5B), which we attributed to the downregulations of the *sitA* and *uvrY* genes, which are responsible for oxidative stress regulation. Unregulated ROS accumulation leads to mitigation of virulence factors by pleotropic reactions with lipids, enzymes, and nucleic acids hindering metabolic functions (Fang, 2004).

As regards the pathogenesis of UTI, motility is used by UPEC to evade human defense systems and infect new sites in bladder lumen or ascend to kidneys (Lane et al., 2007). Bacterial swarming motility, like swimming motility, is achieved by rotating flagella and secreting biosurfactants that reduce surface tension and enable the coordinated movement of cells (Kearns, 2010). Both swarming and swimming motility were inhibited by the three chloroindoles (Figure 3), which may have been due to the significant downregulations of *fliC* (Figure 6), which encodes for flagellin subunits, and the *motA* and *motB* chemotaxis genes, which control cell movements *via* signal transduction (Kalir et al., 2001).

Curli aids cell surface adhesion, cell aggregation, and biofilm formation and is essential for bacterial interactions with serum proteins like fibronectin, laminin, and plasminogen and with extracellular matrix (Barnhart and Chapman, 2006). As evidenced by our Congo-red agar assay results, 4CI, 5CI, and 5CMI inhibited curli and significantly downregulated the ternion of curli genes (Figure 4). *csgA* encodes for the major subunit of curli, whereas *csgB* nucleates CsgA into curli fibers and *csgG* encodes for outer membrane protein. The chloroindoles appeared to repress the *csgAB* operon rather than the *csgDEFG* operon, which would partially explain disruption of curli fiber formation and the observed wrinkled morphology of the UPEC cells (Figures 4, 6).

Indole acts as a signaling molecule and is responsible for resistance to several drugs and the efflux mechanisms deployed by *E. coli* (Lee et al., 2007). Innate indole concentrations were reduced by chloroindole treatments (Figure 5A), which suggested that chloroindoles might competitively inhibit indole production or compete with indole as signaling molecules. Cell surface hydrophobicity favors bacterial adhesion to biotic and abiotic surfaces such as medical devices like implants and catheters (Doyle, 2000), and 4CI, 5CI, and 5CMI all reduced cell surface hydrophobicity (Figure 5C) and thus would be expected to inhibit bacterial colonization of urinary catheters. UPEC employs several fimbriae as part of its virulome, which increases the likelihoods of colonization and persistence. Type-1 fimbriae bind to glycoprotein receptors like uroplakin and aid bladder colonization (Mulvey et al., 1998). On the other hand, P-fimbriae are associated with pyelonephritis and bind to GbO4 isoreceptor in human kidneys, whereas S-fimbriae bind to *α*-sialyl-2,3-*β*-galactoside-containing receptors and aid attachment to endothelial cells (Lane and Mobley, 2007). The three chloroindoles downregulated all fimbriae genes studied (Figure 6), which are essential components of the bacterial adherence virulome, and thereby prevented bacteria switching to an alternative pilus system.

The two-component BarA-UvrY system is responsible for an assortment of important metabolic processes in *E.coli* like the transcriptions of genes, type-1 and pap fimbriae production, motility, extracellular polymeric substance production, biofilm formation, resistance to hydrogen peroxide hypersensitivity (Pernestig et al., 2001; Herren et al., 2006), and regulation of the carbon storage regulatory system (*csr*; Mitra et al., 2013). In the present study, 4CI, 5CI, and 5CMI downregulated the *uvrY* gene and significantly downregulated the *csrA* gene (Figure 6). This observation contrasts to their effects on consensus *csrA* gene levels, which were upregulated to limit biofilm formation and colonization by switching to a more persistent state, which favors infection propagation and bacterial survival. We assert bacterial *csrA* gene activity features only after the stationary phase and that its role in biofilm formation and maturation is minor as compared with other factors like fimbriae, pili, curli, motility, and the two-component BarA-UvrY system as evidenced by two previous studies (Jackson et al., 2002; Mitra et al., 2013).

**Figure 10:**
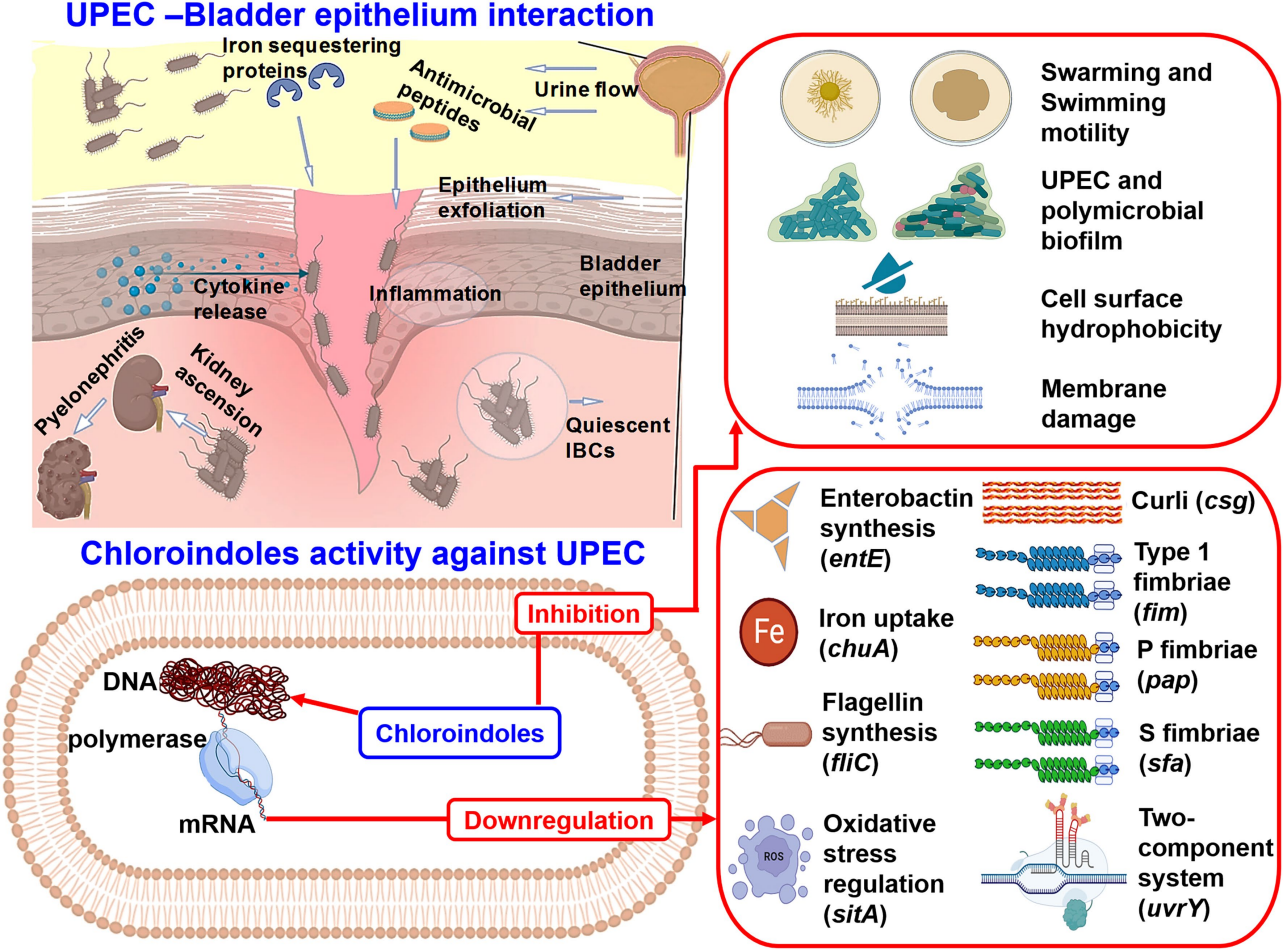
Graphical illustration of the pathogenesis of UPEC, host immune responses, and the antibiofilm and antivirulence activities of 4-chloroindole, 5-chloroindole, and 5-chloro 2-methyl indole against UPEC.

As *uvrY* is a key regulator in UPEC biofilm formation, we believe that downregulation of this gene plausibly explains the actions of chloroindoles (Figure 6). This is partially supported by another study in which allicin decreased biofilm formation, adhesion ability, and motility by downregulating *uvrY* (Yang et al., 2016). A mutant study is required on the *uvrY* gene to confirm this hypothesis. A graphical representation of the pathogenesis of UPEC, host immune responses and the antibiofilm and antivirulence activities of the three chloroindoles is presented in Figure 10.

Chloroindoles also inhibited biofilm formation by other nosocomial pathogens like *S. aureus*, *P. aeruginosa*, *C. albicans*, and *A. baumannii* (Figures 8A–D). We think chloroindoles will have a similar effect on other clinical isolates of UPEC as well. Polymicrobial bacteremia infections are posing an increasing medical challenge and contribute to antimicrobial recalcitrance and virulence. Furthermore, polymicrobial biofilm formation results in greater persistence and pathogenicity (Harriott and Noverr, 2009). We found chloroindoles inhibited UPEC, *S. aureus*, and *C. albicans* polymicrobial biofilm formation (Figure 8E). Interestingly, SEM analysis showed only *S. aureus* cells persisted after chloroindole treatment (Figure 9C). This study further attests to the impact of *S. aureus* in the pathogenesis of polymicrobial infections, in which *S. aureus* benefits from enhanced resistance to antimicrobials (Nair et al., 2014).

3D-QSAR analysis indicated that substitutions at the fourth and fifth positions of the indole moiety increased antimicrobial activity and that substitution at the seventh position was unfavorable (Figure 7B). These positional effects may be due to the influence halogen substitution has on the electronic structure of the indole scaffold, such as on the σ-hole region of low electron density and the dipole that permits binding to lone pair acceptors and donors of the receptors altering ligand binding sterics with its bulk (Bradley et al., 2020). ADME profiling of 4CI, 5CI, and 5CMI predicted them to be non-carcinogenic to mice, to have minimal acute fish toxicities, and not to violate Lipinski’s rule of five (Supplementary Table S6). 5-Chloroindole has also been reported to be non-cytotoxic to the MN9D mouse dopaminergic neuronal cell line (Kholodar et al., 2021).

## Conclusion

This is the first study to report the antibacterial and antibiofilm activities of chloroindoles against UPEC. Three chloroindoles namely, 4-chloroindole, 5-chloroindole, and 5-chloro 2-methyl indole, were found to have bactericidal, anti-adherent, and virulence factor inhibitory effects on UPEC. Antimicrobial activities were attributed to unregulated ROS production rendered probably by the downregulations of oxidative stress genes, especially the *uvrY* gene. 3-D QSAR analysis revealed the fourth and fifth positions of the indole moiety to be favorable for functional groups for antimicrobial activity and the seventh position as unfavorable. All three selected chloroindoles exhibited rapid killing activity on-par with some common antibiotics and potently killed other nosocomial pathogens and prevented polymicrobial biofilm formation. Furthermore, the ADME profiles of the three derivatives indicated they have suitable drug-likeness properties. We believe our results support the notion that the three selected indole derivatives, that is, 4-chloroindole, 5-chloroindole, and 5-chloro 2-methyl indole, have therapeutic potentials as broad-spectrum drugs against UTIs.

## Data Availability Statement

The original contributions presented in the study are included in the article/Supplementary Material, further inquiries can be directed to the corresponding author.

## Author Contributions

J-HL and JL: conceptualization, project administration, and funding acquisition. BB and J-HL: methodology. BB: software. BB, J-HL, and JL: validation and writing the manuscript. JL: resources and supervision. All authors contributed to the article and approved the submitted version.

## Funding

This research was supported by the Basic Science Research Program of the National Research Foundation of Korea (NRF) funded by the Ministry of Education (grant no. 2021R1I1A3A04037486 to J-HL), the NRF funded by the Korean Government (MSIT; grant no. 2021R1A2C1008368), and by the Priority Research Center Program of the NRF funded by the Ministry of Education (grant no. 2014R1A6A1031189).

## Conflict of Interest

The authors declare that the research was conducted in the absence of any commercial or financial relationships that could be construed as a potential conflict of interest.

## Publisher’s Note

All claims expressed in this article are solely those of the authors and do not necessarily represent those of their affiliated organizations, or those of the publisher, the editors and the reviewers. Any product that may be evaluated in this article, or claim that may be made by its manufacturer, is not guaranteed or endorsed by the publisher.

## Footnotes

^1^ https://imagej.nih.gov/

^2^ https://preadmet.qsarhub.com/

^3^ https://www.molinspiration.com/

^4^ http://www.way2drug.com/gusar/
