# Supplementary material for: Antibiofilm and Antimicrobial Activities of Chloroindoles Against Uropathogenic Escherichia coli
Source: Front Microbiol. 2022 Jun 16;13:872943. doi: 10.3389/fmicb.2022.872943 (PMC9244173; doi:10.3389/fmicb.2022.872943)
Supplement: Supplementary file 1 [file Data_Sheet_1.docx]

**Supplementary Material**

**Broad-spectrum antibiofilm activities of chloroindoles against uropathogenic *Escherichia coli* and other nosocomial pathogens**

Bharath Reddy Boya^1^, Jin-Hyung Lee^1^, Jintae Lee*

School of Chemical Engineering, Yeungnam University, 280 Daehak-Ro, Gyeongsan, 38541,

Republic of Korea

^1^B.R.B. and J.-H.L. contributed equally to this work.

*Corresponding author

E-mail: jtlee@ynu.ac.kr; Tel: +82-53-810-2533; Fax: +82-53-810-4631

**Table S1.** MICs and % biofilm inhibition of 83 indole derivatives against UPEC at concentrations 20 and 100 µg/ml. MICs (minimum inhibitory concentrations) were defined as the lowest concentrations that prevented bacterial growth.

| **Indole Derivative** | **MIC** | **% biofilm inhibition**  **20 µg/ml** | **% biofilm inhibition 100 µg/ml** |
| --- | --- | --- | --- |
| 4 Iodoindole | 100 | 74 | 99 |
| 5 Iodoindole | 100 | 53 | 98 |
| 6 Iodoindole | 100 | 8 | 84 |
| 7 Iodoindole | 200 | 31 | 68 |
| 4 Fluoroindole | 200 | 78 | 81 |
| 5 fluoroindole | 200 | 31 | 78 |
| 6 Fluoroindole | 200 | 72 | 78 |
| 7 Fluoroindole | 400 | 52 | 68 |
| 4 Chloroindole | 75 | 72 | 98 |
| 5 Chloroindole | 75 | 66 | 99 |
| 6 Chloroindole | 100 | 6 | 79 |
| 7 Chloroindole | 400 | 29 | 65 |
| 4 Bromoindole | 50 | 68 | 97 |
| 5 Bromoindole | 75 | 79 | 98 |
| 6 Bromoindole | 200 | 28 | 88 |
| 7 Bromoindole | 200 | 28 | 91 |
| 7-Hydroxyindole (7HI) | >400 | 30 | 55 |
| 3-Indoleacetic acid (I3Ac) | >400 | 7 | 36 |
| Indole-3-pyruvic acid (I3PYA) | >400 | 29 | 52 |
| 5-(Trifluoromethoxy)Indolin-2,3-dione 97% (5TFMOI23D) | >400 | 58 | 69 |
| 5-Fluorooxindole 98% (5FOI) | >400 | 24 | 42 |
| 5-Fluoroindoline-2,3-dione 98% (5FI23D) | 400 | 26 | 59 |
| 7-Fluoroindoline-2,3-dione 98% (7FI23D) | 200 | 20 | 37 |
| 6-Trifluoromethylindole (6TFMI) | 200 | 17 | 40 |
| 1-BOC-5-iodoindole (1BOC5II) | 200 | 24 | 22 |
| 7-Fluoro-5-iodoindole-3-carboxaldehyde (7F5II3C) | 400 | 44 | 80 |
| Indole-3-carboxaldehyde 97% (I3CA) | >400 | 47 | 66 |
| 1-Methylindole-3-carboxaldehyde (1MI3C) | >400 | 24 | 77 |
| 2-Methylindole-3-carboxaldehyde (2My3C) | >400 | 57 | 69 |
| 7-Methylindole-3-carboxaldehyde (7MYI3C) | 400 | 55 | 75 |
| 1-BOC-7-methylindole 95% (1BOC7MI) | >400 | 24 | 69 |
| 3-Methylindole 98% | 400 | 51 | 76 |
| 7-Methylindole (7MYI) | 400 | 52 | 69 |
| 1,2-Dimethylindole (1,2-DMI) | 200 | 39 | 36 |
| 4-Benzyloxyindole (4BOI) | 200 | 33 | 43 |
| 5-Benzyloxyindole (5BOI) | 200 | 20 | 49 |
| 6-Benzyloxyindole (6BOI) | >400 | 37 | 39 |
| 7-Benzyloxyindole 99% (7BOI) | 200 | 27 | 29 |
| Indole-3-carboxylic acid (I3CA) | >400 | 9 | 33 |
| Indole-7-carboxylic acid 98% (I7CA) | >400 | 3 | 31 |
| Indole-3-butyric acid (I3BA) | >400 | 14 | 7 |
| Methyl indole-7-carboxylate 99% (MI7C) | >400 | 14 | 35 |
| 3-Indoleacetonitrile (3IAN) | >400 | 14 | 20 |
| Indole-3-acetamide 98% (I3Act) | >400 | 1 | 12 |
| Indole-3-carbinol (I3C) | >400 | 6 | 2 |
| 7-Azaindole 98% (7Aza) | >400 | 6 | 5 |
| 5-bromo-3-iodo-7-azaindole (5B3I7AI) | >400 | 8 | 50 |
| 5-Fluoro-4-iodo-1H-pyrrolo[2,3-b]pyridine (5F4IP2,3P) | >400 | 11 | 31 |
| 5-Iodoindolin-2-one (5I2O) | >400 | 48 | 76 |
| 6-Iodo-2,3-dihydro-1H-indole(6-I-23-DH1H-I) | 400 | 31 | 55 |
| 7-Formylindole 99% (7FYI) | 400 | 35 | 60 |
| 7-Methoxyindole 97% (7MOI) | >400 | 1 | 11 |
| 7-Nitroindole 98% (7NI) | 400 | 59 | 75 |
| 3,3`-Methylenebisindole (3,3`MBSI) | 200 | 17 | 43 |
| 5-Methoxyisatin 98% (5MOIs) | 400 | 21 | 56 |
| 5-Methylisatin 98% (5MYIs) | 200 | 25 | 75 |
| 5-Nitroisatin 95% (5NIs) | 400 | 27 | 58 |
| 7-Chloroisatin 97% (7ClIs) | 400 | 23 | 56 |
| 5-Chloroisatin (5ClIs) | 200 | 23 | 62 |
| 5-Bromoisatin 95% (5Bis) | 400 | 26 | 58 |
| Indole-3-propionic acid (I3PA) | >400 | 10 | 18 |
| 2,5-Dimethylindole (2,5DYI) | >400 | 18 | 42 |
| 5-Amino-2-Methylindole (5A2MYI) | >400 | 26 | 50 |
| 2-Methylindole-3-acetic acid (2MI3Ac) | >400 | 5 | 6 |
| 5-Fluoro-2-methylindole 95% (5F2MI) | >400 | 46 | 85 |
| 5-Formylindole 99% (5FYI) | >400 | 35 | 55 |
| 5-Fluoro-1H-Indazole (5FIHI) | >400 | 3 | 38 |
| 3-(2-Hydroxyethyl)indole (3-(2,HE)I) | >400 | 14 | 31 |
| 4,5,6,7-Tetrahydro-1H-Indazole HCl 98% (4567THI) | >400 | 10 | 25 |
| 5-Chloro-2-Methylindole (5CMI) | 75 | 64 | 95 |
| 6-Formylindole (6FYI) | >400 | 37 | 51 |
| 2-Oxindole (2OI) | >400 | 12 | 28 |
| 4-Formylindole (4FYI) | >400 | 24 | 62 |
| 5-Fluoro-4-Iodo-1H-pyrrolo-5-Carboxylic acid (5F4IP2,3P) | >400 | 37 | 66 |
| 5-Methylindole | 400 | 33 | 56 |
| Melatonin | >400 | 35 | 29 |
| 7-(Trifluoromethyl) indoline 2,3-di one | 400 | 44 | 55 |
| Isatin | 200 | 32 | 74 |
| 7-trifluoromethyl isatin | 400 | 42 | 46 |
| 7-methyl-1H-isatin | 400 | 25 | 68 |
| 7-fluoroisatin | 400 | 40 | 76 |
| 1H indole 2-carbaldehyde | 400 | 29 | 54 |
| 7-methyl-1H-indole 2,3-di one | 400 | 25 | 61 |
| 2,3-dimethylindole | 200 | 29 | 67 |
| 6-Iodo-2,3-dihydro-1H-indole hydrochloride | >400 | 15 | 30 |
| 5,6-difluoroisatin | 200 | 32 | 62 |
| INDOLE | >400 | 3 | 5 |

**Table S2.** 18 Genes used in the study associated with UPEC, their function, and primer sequences. *rrsG* was used as the housekeeping gene

| **Gene** | **Function** | **Primer** |
| --- | --- | --- |
| *csgA* | Curli subunit | F -5'-GGG CTC AGA TGA CAG CTC AAT C-3'  R- 5'-GCC GTT CCA CTG ATC AAG AGT AG-3' |
| *csgB* | Curli subunit | F - 5'-CAT AAT TGG TCA AGC TGG GAC TAA-3'  R-5'-GCA ACA ACC GCC AAA AGT TT-3' |
| *csgG* | Curli subunit | F -5'-TGG TTG CCG TCA TGT TAC TGA-3'  R-5'-CGG TCT GGC GGC TTC TTT A-3' |
| *fimA* | Type 1 fimbriae subunit | F - 5'-ACT CTG GCA ATC GTT GTT CTG TCG-3'  R-5'-ATC AAC AGA GCC TGC ATC AAC TGC-3' |
| *fimH* | Type 1 fimbriae adhesin | F -5'-GAT GCG GGC AAC TCG ATT-3'  R-5'-CCC TGC GCG GGT GAA-3' |
| *motA* | Motility | F -5'-ACA GGT AGC GCG TTC TCA CT-3'  R-5'-AGC GTG GAT AAA CCG ATA CG-3' |
| *motB* | Motility | F -5'-GCG TTA CGT CCA CAT CTC AA-3'  R-5'-ATG TCG CGC ATA TAG GGT TC-3' |
| *fliC* | Flagellin protein | F -5'-ACA GCC TCT CGC TGA TCA CTC AAA-3'  R-5'-GCG CTG TTA ATA CGC AAG CCA GAA-3' |
| *papA* | Pyelonephritis associated protein fimbriae | F -5'-GTG CCT GCA GAA AAT GCA GAT-3'  R-5'-CCC GTT TTC CAC TCG AAT CA-3' |
| *papG* | Pyelonephritis associated protein fimbriae adhesin | F -5'-GGG AGG GAA TGT GGT GAT TAC TC-3'  R-5'-CGG GCG CCA CGA AGT-3' |
| *sitA* | Iron acquisition and oxidative stress regulator | F -5’-TCC ACC GCT TTT ACC AGC ATC T-3’  R-5’-TCT GGC GAC ATC CAT GCA T-3’ |
| *sfaA* | S.fimbrial adhesin | F -5'-CCG TTC CAG GCT CGC TAT ATT-3'  R-5'-CGG CGT TGG CTG TAC CA-3' |
| *sfaS* | S.fimbrial adhesin | F -5'-TCT CAC CGG ATG CCA GAA TAT-3'  R-5'-GCC CAT CCG CAG TAC CAC TA-3' |
| *chuA* | Heme uptake and transport in UTI | F -5’-TGC CAA CGT TTG CTT TTG CT-3’  R-5’-TCA GGA GCG GAA GTG TCG ATA -3’ |
| *entE* | Enterobactin synthase -Iron starvation response (siderophore) | F -5’-ATT GAA CCG GCG TTA CTG ATT G-3’  R-5’-CAC CAC GCG AAT GGA AGA AT-3’ |
| *uvrY* | Two-component system regulation | F -5’-TCA GAC AAA CTG GCA AAT GG-3’  R-5’-CTA TTC AGG GCA GCG TTA CA-3’ |
| *csrA* | Carbon metabolism, biofilm attenuation and dispersal | F -5’-CCT GGA TAC GCT GGT AGA T-3’  R-5’-TCG TCG AGT TGG TGA GAC-3’ |
| *rrsG* | Housekeeping (endogenous control) | F -5'-CCA GGG CTA CAC ACG TGC TA-3'  R-5'-TCT CGC GAG GTC GCT TCT-3' |

**Figure S1-** Rapid killing activity of indoles in comparison to common antibiotics in UPEC. At the indicated time points, aliquots of treated cells were plated and CFUs were enumerated. Error bars represent standard deviation.

**Table S3.** 5 QSAR PLS factor models built based on the 73 indole derivatives. PLS factor model 5 was chosen based on the low S.D, and high R^2, stability and F ratio.

| **Factor** | **S.D** | **R^2** | **R^2 CV** | **R^2 scramble** | **Stability** | **F** | **P** | **RMSE** | **Q^2** | **Pearson-r** |
| --- | --- | --- | --- | --- | --- | --- | --- | --- | --- | --- |
| 1 | 0.2651 | 0.1496 | 0.0767 | 0.112 | 0.989 | 9.2 | 0.00386 | 0.26 | -1.2601 | -0.5845 |
| 2 | 0.1813 | 0.61 | 0.4121 | 0.3644 | 0.885 | 39.9 | 3.72E-11 | 0.21 | -0.5009 | 0.2861 |
| 3 | 0.1338 | 0.7918 | 0.552 | 0.5335 | 0.857 | 63.4 | 4.68E-17 | 0.22 | 0.5375 | 0.3769 |
| 4 | 0.1038 | 0.877 | 0.5903 | 0.6566 | 0.821 | 87.4 | 1.13E-21 | 0.21 | -0.4926 | 0.4471 |
| 5 | 0.093 | 0.9033 | 0.6185 | 0.7207 | 0.809 | 89.7 | 3.70E-23 | 0.21 | -0.496 | 0.517 |

**Table S4.** QSAR activity prediction of the 73 indole derivatives. QSAR set represents training and test set 70:30 ramdomization of 73 indole derivatives. PLS factor 5 was chosen as the best model based on the predicted pMIC values

| **Ligand Name** | **QSAR Set** | **Activity pMIC [-log(MIC)]** | **PLS Factors** | **Predicted Activity (Predicted pMIC)** |
| --- | --- | --- | --- | --- |
| 4-bromoindole | training | -2.699 | 1 2 3 4 5 | -3.38604 -3.21238 -2.98998 -2.86752 -2.84216 |
| 4-Chloroindole | training | -2.875 | 1 2 3 4 5 | -3.38714 -3.225 -3.04539 -2.94621 -2.92669 |
| 5-bromoindole | training | -2.875 | 1 2 3 4 5 | -3.37937 -3.09406 -2.96683 -2.92308 -2.92457 |
| 5-Chloroindole | training | -2.875 | 1 2 3 4 5 | -3.38136 -3.1197 -3.01741 -2.96774 -2.96466 |
| 5-Chloro 2methyl indole | training | -2.875 | 1 2 3 4 5 | -3.37683 -3.07802 -3.00487 -2.9618 -2.93908 |
| 4-Iodo indole | training | -3 | 1 2 3 4 5 | -3.38547 -3.21498 -2.95108 -2.83118 -2.81606 |
| 5-Iodo indole | training | -3 | 1 2 3 4 5 | -3.37906 -3.08846 -2.96311 -2.93597 -2.95079 |
| 6-Iodo indole | training | -3 | 1 2 3 4 5 | -3.3849 -3.17691 -3.16173 -3.16284 -3.16788 |
| 1,2 Dimethyl Indole | training | -3.301 | 1 2 3 4 5 | -3.4002 -3.37374 -3.42919 -3.34114 -3.25555 |
| 1-BOC-5-Iodoindole | test | -3.301 | 1 2 3 4 5 | -3.40233 -3.44635 -3.47573 -3.24843 -3.0885 |
| 2,3 Dimethyl Indole | training | -3.301 | 1 2 3 4 5 | -3.39604 -3.4212 -3.48394 -3.47327 -3.4795 |
| 4-Fluoroindole | test | -3.301 | 1 2 3 4 5 | -3.38935 -3.24527 -3.17509 -3.12379 -3.12149 |
| 4-Benzoyl indole | test | -3.301 | 1 2 3 4 5 | -3.41015 -3.31979 -3.06966 -3.15925 -3.11366 |
| 5-Fluoroindole | training | -3.301 | 1 2 3 4 5 | -3.3857 -3.18593 -3.14645 -3.11804 -3.12723 |
| 5,6 Difluoroisatin | training | -3.301 | 1 2 3 4 5 | -3.63863 -3.56967 -3.53487 -3.48027 -3.45813 |
| 5-Benzoylindole | training | -3.301 | 1 2 3 4 5 | -3.40048 -3.3219 -3.17926 -3.36488 -3.27745 |
| 5-Chloroisatin | test | -3.301 | 1 2 3 4 5 | -3.6396 -3.56898 -3.54174 -3.49938 -3.49197 |
| 5-Methylisatin | training | -3.301 | 1 2 3 4 5 | -3.63919 -3.56284 -3.52472 -3.47243 -3.45758 |
| 6-Bromoindole | training | -3.301 | 1 2 3 4 5 | -3.38597 -3.19242 -3.19081 -3.19685 -3.20895 |
| 6-Fluoroindole | training | -3.301 | 1 2 3 4 5 | -3.38912 -3.23386 -3.24927 -3.25261 -3.27102 |
| 6-(trifluoromethyl)indole | training | -3.301 | 1 2 3 4 5 | -3.38532 -3.19159 -3.21171 -3.24143 -3.26175 |
| 7-Bromoindole | training | -3.301 | 1 2 3 4 5 | -3.39169 -3.30584 -3.44453 -3.46205 -3.44955 |
| 7-Iodoindole | training | -3.301 | 1 2 3 4 5 | -3.39145 -3.3055 -3.44529 -3.45287 -3.42819 |
| 7-Fluoroisatin | test | -3.301 | 1 2 3 4 5 | -3.63733 -3.58981 -3.59251 -3.58595 -3.58887 |
| Isatin | test | -3.301 | 1 2 3 4 5 | -3.63674 -3.58745 -3.58748 -3.57331 -3.57292 |
| Indole-2 carboxaldehyde | training | -3.602 | 1 2 3 4 5 | -3.38948 -3.26581 -3.39101 -3.45202 -3.49278 |
| 3-Methylindole | test | -3.602 | 1 2 3 4 5 | -3.40057 -3.46288 -3.49649 -3.47921 -3.50508 |
| 5-Bromoisatin | training | -3.602 | 1 2 3 4 5 | -3.6401 -3.56974 -3.54464 -3.50411 -3.5 |
| 5-Fluoroisatin | training | -3.602 | 1 2 3 4 5 | -3.63812 -3.5749 -3.55514 -3.52077 -3.51265 |
| 5-Methoxyisatin | training | -3.602 | 1 2 3 4 5 | -3.64108 -3.59167 -3.60033 -3.58561 -3.59107 |
| 5-Nitroisatin | training | -3.602 | 1 2 3 4 5 | -3.63759 -3.59149 -3.60741 -3.60088 -3.60606 |
| 6-Iodoindoline | training | -3.602 | 1 2 3 4 5 | -3.62331 -3.65071 -3.72146 -3.66878 -3.63966 |
| 7-Chloroindole | test | -3.602 | 1 2 3 4 5 | -3.3921 -3.30871 -3.44539 -3.47153 -3.47135 |
| 7-Fluoroindole | training | -3.602 | 1 2 3 4 5 | -3.39216 -3.29297 -3.38233 -3.41024 -3.43438 |
| 7-Methylindole | training | -3.602 | 1 2 3 4 5 | -3.39238 -3.31503 -3.46083 -3.48951 -3.49064 |
| 7-Chloroisatin | training | -3.602 | 1 2 3 4 5 | -3.63807 -3.57588 -3.56957 -3.59431 -3.61158 |
| 7-fluoro-5-iodo-1H-indole-3-carbaldehyde | training | -3.602 | 1 2 3 4 5 | -3.39686 -3.47514 -3.46465 -3.46993 -3.51856 |
| Indole-7-carboxaldehyde | training | -3.602 | 1 2 3 4 5 | -3.39431 -3.35576 -3.58708 -3.67011 -3.68671 |
| 7-Methylindole-3Carboxaldehyde | test | -3.602 | 1 2 3 4 5 | -3.40919 -3.66981 -3.86588 -3.89306 -3.91898 |
| 7-methylindoline 2,3-dione | training | -3.602 | 1 2 3 4 5 | -3.63847 -3.57533 -3.56907 -3.60588 -3.62923 |
| 7-Nitroindole | training | -3.602 | 1 2 3 4 5 | -3.39524 -3.34056 -3.56264 -3.67422 -3.70508 |
| Indole | test | -3.602 | 1 2 3 4 5 | -3.39117 -3.26108 -3.28583 -3.27983 -3.29495 |
| 7-Fluoroisatin | training | -3.602 | 1 2 3 4 5 | -3.63733 -3.58981 -3.59251 -3.58595 -3.58887 |
| 1-(3-(2-isopropyl-5-methylphenoxy)propyl)-7-methylindoline-2,3-dione | training | -3.602 | 1 2 3 4 5 | -3.5598 -3.701 -3.38892 -3.64279 -3.64623 |
| 1-BOC-7-Methylindole | training | -3.699 | 1 2 3 4 5 | -3.41549 -3.66736 -3.9553 -3.77813 -3.60294 |
| 1-Methylindole-3-Carboxaldehyde | training | -3.699 | 1 2 3 4 5 | -3.42154 -3.7702 -3.84677 -3.75063 -3.70947 |
| 2-Oxindole | training | -3.699 | 1 2 3 4 5 | -3.63113 -3.62932 -3.69742 -3.7008 -3.71424 |
| 2-Methylindole-3-Acetic acid | test | -3.699 | 1 2 3 4 5 | -3.41119 -3.75245 -3.75459 -3.69045 -3.72547 |
| 2-Methylindole-3-carboxaldehyde | test | -3.699 | 1 2 3 4 5 | -3.40345 -3.57418 -3.67833 -3.67744 -3.6977 |
| Tryptophol | training | -3.699 | 1 2 3 4 5 | -3.41239 -3.72515 -3.69068 -3.62707 -3.68092 |
| 3-Indoleacetonitrile | training | -3.699 | 1 2 3 4 5 | -3.41261 -3.73189 -3.68586 -3.62984 -3.68767 |
| 5-Amino-2-Methylindole | training | -3.699 | 1 2 3 4 5 | -3.39054 -3.29438 -3.49647 -3.63782 -3.65985 |
| 5-Bromo-3-Iodod-7-Azaindole | test | -3.699 | 1 2 3 4 5 | -3.39768 -3.35113 -3.28671 -3.26314 -3.30032 |
| 5-Fluoroxindole | training | -3.699 | 1 2 3 4 5 | -3.63252 -3.61677 -3.66508 -3.64827 -3.65397 |
| 5-Iodoindolin-2-one | training | -3.699 | 1 2 3 4 5 | -3.6349 -3.61439 -3.66207 -3.64253 -3.65505 |
| 5-(Trifluoromethoxy) Isatin | training | -3.699 | 1 2 3 4 5 | -3.6424 -3.60625 -3.64961 -3.66319 -3.68926 |
| 6-Benzoylindole | training | -3.699 | 1 2 3 4 5 | -3.44406 -3.69708 -3.6039 -3.86807 -3.72603 |
| 6-Iodoindoline | training | -3.699 | 1 2 3 4 5 | -3.62331 -3.65071 -3.72146 -3.66878 -3.63966 |
| 7-Azaindole | test | -3.699 | 1 2 3 4 5 | -3.39821 -3.27852 -3.35221 -3.37297 -3.40363 |
| 7-Hydroxyindole | training | -3.699 | 1 2 3 4 5 | -3.39471 -3.32329 -3.4947 -3.58837 -3.64266 |
| 7-Methoxyindole | training | -3.699 | 1 2 3 4 5 | -3.4372 -3.70305 -3.59942 -3.6817 -3.63045 |
| 7-(Trifluoromethyl)Isatin | training | -3.699 | 1 2 3 4 5 | -3.63894 -3.57169 -3.56693 -3.61723 -3.64727 |
| 4,5,6,7-Tetrahydroindazole | test | -3.699 | 1 2 3 4 5 | -3.42814 -3.41923 -3.39998 -3.36802 -3.36146 |
| Indole-3-acetic acid | training | -3.699 | 1 2 3 4 5 | -3.41572 -3.79413 -3.76714 -3.69639 -3.75106 |
| Indole -3-Acetamide | training | -3.699 | 1 2 3 4 5 | -3.41654 -3.79746 -3.77895 -3.71511 -3.75501 |
| Indole -3-Butyric acid | training | -3.699 | 1 2 3 4 5 | -3.41076 -3.71736 -3.56625 -3.56475 -3.65022 |
| Indole-3-Carbinol | training | -3.699 | 1 2 3 4 5 | -3.41047 -3.66518 -3.74691 -3.74476 -3.78652 |
| Indole-3-carboxaldehyde | test | -3.699 | 1 2 3 4 5 | -3.40799 -3.61586 -3.69087 -3.68338 -3.72328 |
| Indole-3-Carboxylic acid | test | -3.699 | 1 2 3 4 5 | -3.40943 -3.64331 -3.72038 -3.71967 -3.75667 |
| 3-Indoleprepionic acid | test | -3.699 | 1 2 3 4 5 | -3.41093 -3.70372 -3.61042 -3.56105 -3.61462 |
| Indole-3-Pyruvic acid | training | -3.699 | 1 2 3 4 5 | -3.41554 -3.78607 -3.73633 -3.6816 -3.73245 |
| Indole-7-carboxylic acid | test | -3.699 | 1 2 3 4 5 | -3.44041 -3.77666 -3.71375 -3.75347 -3.71664 |
| Melatonin | test | -3.699 | 1 2 3 4 5 | -3.41246 -3.77513 -3.63074 -3.72884 -3.74782 |
| Methyl Indole-7-carboxylate | training | -3.699 | 1 2 3 4 5 | -3.4418 -3.81928 -3.65158 -3.6885 -3.64587 |

**Table S5.** External validation of QSAR model with 10 indole derivatives with varying MICs.The highlighted red values represent PLS factor 5 which was the chosen model for QSAR visualization.

| **Ligand Name** | **Activity** | **PLS Factors** | **Predicted Activity** |
| --- | --- | --- | --- |
| 6-Chloroindole | -2.699 | 1 2 3 4 5 | -3.38604 -3.21238 -2.98998 -2.86752 -2.84216 |
| 7-Benzoylindole | -3.301 | 1 2 3 4 5 | -3.63919 -3.56284 -3.52472 -3.47243 -3.45758 |
| 5-Methylindole | -3.602 | 1 2 3 4 5 | -3.38948 -3.26581 -3.39101 -3.45202 -3.49278 |
| 2,5 Dimethylindole | -3.699 | 1 2 3 4 5 | -3.41549 -3.66736 -3.9553 -3.77813 -3.60294 |
| 5-Fluoro-2-Methyindole | -3.699 | 1 2 3 4 5 | -3.44406 -3.69708 -3.6039 -3.86807 -3.72603 |
| 5-Fluoro-4-iodo-1H-pyrrolo[2,3-b]pyridine | -3.699 | 1 2 3 4 5 | -3.4372 -3.70305 -3.59942 -3.6817 -3.63045 |
| 5-Fluoroindazole | -3.699 | 1 2 3 4 5 | -3.41572 -3.79413 -3.76714 -3.69639 -3.75106 |
| Indole-6-carboxaldehyde | -3.699 | 1 2 3 4 5 | -3.41554 -3.78607 -3.73633 -3.6816 -3.73245 |
| Indole-4-carboxaldehyde | -3.699 | 1 2 3 4 5 | -3.39821 -3.27852 -3.35221 -3.37297 -3.40363 |
| Indole-5-carboxaldehyde | -3.699 | 1 2 3 4 5 | -3.63113 -3.62932 -3.69742 -3.7008 -3.71424 |

**Table S6.** ADME profile of the three chloroindoles selected in the study. The profile is an amalgamation of the ADME properties of the chloroindoles from the online webservers, viz. PreADMET, Molinspiration and GUSAR all of which were accessed on 8^th^ September 2021

| **Property** | **4CI** | **5CI** | **5CMI** |
| --- | --- | --- | --- |
| **Lead like rule** | Suitable | suitable | Suitable |
| **Lead like violations** | 0 | 0 | 0 |
| **Lipinski rule of five violations** | 0 | 0 | 0 |
| **Plasma protein binding** | 100% | 54.64% | 32.297361 |
| **blood brain barrier permeability** | 10.3009 | 10.3009 | 10.7545 |
| **Skin absorption** | -2.54009 | -2.51146 | -2.52169 |
| **Human intestinal absorption** | 100% | 100% | 100% |
| **Caco 2** | 55.0337 | 55.0337 | 55.6688 |
| **Mouse carcinogenicity** | none | none | none |
| **Acute fish toxicity (medaka)** | 0.0400662 | 0.0400662 | 0.0257724 |
| **Acute fish toxicity (minnow)** | 0.027543 | 0.027543 | 0.0258809 |
| **in vitro hERG inhibition** | medium | medium | medium |
| **miLogP** | 2.79 | 2.81 | 3.04 |
| **mol volume** | 126.56 | 126.56 | 143.12 |
| **tpsa** | 15.79 | 15.79 | 15.79 |
| **GPCR ligand** | -0.63 | -0.57 | -0.62 |
| **Ion channel modulator** | -0.08 | 0 | -0.14 |
| **Kinase inhibitor** | -0.59 | -0.33 | -0.48 |
| **Nuclear receptor ligand** | -0.95 | -0.95 | -0.92 |
| **Protease inhibitor** | -1.3 | -1.11 | -1.08 |
| **Enzyme inhibitor** | -0.46 | -0.3 | -0.3 |
| **Rat IP LD50 classification** | Class 4 in AD | Class 5 in AD | Class 5 in AD |
| **Rat IV LD50 classification** | Class 4 in AD | Class 4 in AD | Class 4 in AD |
| **Rat oral LD50 classification** | Class 4 in AD | Class 4 in AD | Class 4 in AD |
| **Rat SC LD50 classification** | Class 4 in AD | Class 4 in AD | Class 4 in AD |

**Table S7.** MIC’s of the selected chloroindoles against various nosocomial pathogens. Luria Bertani media was used for *S. aureus* and *P. aeruginosa* whereas tryptic soy broth and potato dextrose broth was used for *A. baumannii* and *C. albicans* respectively. MICs (minimum inhibitory concentrations) were defined as the lowest concentrations that prevented bacterial growth.

| **Compound** |  | ***A. baumannii*** | ***S. aureus*** | ***C. albicans*** | ***P. aeruginosa*** |
| --- | --- | --- | --- | --- | --- |
| 4CI |  | 50µg/ml | 50µg/ml | 50µg/ml | 400µg/ml< |
| 5CI |  | 50µg/ml | 100µg/ml | 100µg/ml | 400µg/ml< |
| 5CMI |  | 100µg/ml | 100µg/ml | 100µg/ml | 400µg/ml< |
